# Supplementary material for: Lowering blood pressure after acute intracerebral haemorrhage: protocol for a systematic review and meta-analysis using individual patient data from randomised controlled trials participating in the Blood Pressure in Acute Stroke Collaboration (BASC)
Source: BMJ Open. 2019 Jul 16;9(7):e030121. doi: 10.1136/bmjopen-2019-030121 (PMC6661570; doi:10.1136/bmjopen-2019-030121)
Supplement: Supplementary data [file bmjopen-2019-030121supp001.pdf]

SUPPLEMENTARY MATERIAL 1: Search Strategy

Medline search strategy

1. exp basal ganglia hemorrhage/ or intracranial hemorrhages/ or cerebral hemorrhage/ or intracranial hemorrhage, hypertensive/ or cerebrovascular disorders/

2. ((brain$ or cerebr$ or cerebell$ or intracerebral or intracran$ or parenchymal or intraparenchymal or intraventricular or infratentorial or supratentorial or basal gangli$ or putaminal or putamen or posterior fossa or hemispher$ or pon$ or lentiform$ or brainstem or cortic$ or cortex$ or subcortic$ or subcortex$) adj5 (h?emorrhag$ or h?ematoma$ or bleed$)).tw

3. ((hemorrhag$ or haemorrhag$) adj6 (stroke$ or apoplex$ or cerebral vasc$ or cerebrovasc$ or cva)).tw

4. (ICH or ICHs or PICH or PICHs).tw

5. 1 or 2 or 3 or 4

6. exp blood pressure/

7. exp hypertension/

8. (blood pressure or bloodpressure).tw

9. ((bp or blood pressure) adj5 (lowering or reduc$)).tw

10. ((strict$ or target$ or tight$ or intens$ or below) adj3 (blood pressure or systolic or diastolic or bp or level$)).tw

11. (hypertension or hypertensive).tw

12. ((manage$ or monitor$) adj3 (hypertension or blood pressure)).tw

13. ((intense or intensive or aggressive or accelerated or profound or radical or severe) adj5 ((bp or blood pressure) adj5 (lowering or reduc$ or decreas$ or decrement or dimin$ or declin$))).tw

14. ((standard or normal or ordinary or guideline or guide line or guideline recommend$ or recommend$ or convention$ or usual or established) adj5 ((bp or blood pressure) adj5 (lowering or reduc$ or decreas$ or decrement or dimin$ or declin$))).tw

15. (antihypertensive adj2 (agent$ or drug$ or medicat$)).tw

16. 6 or 7 or 8 or 9 or 10 or 11 or 12 or 13 or 14 or 15

17. exp nitroglycerin/

18. nitrate.tw

19. glyceryl trinitrate.mp. or GTN.tw. [mp=title, abstract, original title, name of substance word, subject heading word, keyword heading word, protocol supplementary concept word, rare disease supplementary concept word, unique identifier, synonyms]

20. nitric Oxide Donors.tw

21. exp angiotensin-converting enzyme inhibitor/

22. exp ace inhibitor/

23. angiotensin-converting enzyme inhibitor$.mp

24. ace inhibitor$.mp

25. (alacepril or altiopril or benazepril or captopril or ceronapril or cilazapril or delapril or derapril or enalapril or fosinopril or idapril or imidapril or lisinopril or moexipril or moveltipril or pentopril or perindopril or quinapril or ramipril or spirapril or temocapril or randolapril or zofenopril).mp

26. exp Angiotensin Receptor Antagonists/

27. (angiotensin adj3 (receptor antagon$ or receptor block$)).tw

28. arb?.tw

29. (abitesartan or azilsartan or candesartan or elisartan or embusartan or eprosartan or forasartan or irbesartan or losartan or milfasartan or olmesartan or saprisartan or tasosartan or telmisartan or valsartan or zolasartan).mp

30. exp adrenergic beta-antagonists/

31. (beta adj2 (adrenergic? or antagonist? or block$ or receptor?)).tw

32. (acebutolol or alprenolol or amosulalol or arotinolol or atenolol or befunolol or betaxolol or bevantolol or bisoprolol or bopindolol or bucindolol or bucumolol or bufetolol or bufuralol or bunitrolol or bupranolol or butofilolol or carazolol or carteolol or carvedilol or celiprolol or cetamolol or cloranolol or cyanopindolol or deacetylmetipranolol or dihydroalprenolol or dilevalol or epanolol or esmolol or indenolol or iodocyanopindolol or labetalol or landiolol or levobunolol or mepindolol or metoprolol or metipranolol or moprolol or nadolol or nadoxolol

or nebivolol or nifenalol or nipradilol or oxprenolol or penbutolol or pindolol or practolol or pronethalol or propranolol or proxodolol or sotalol or sulfinalol or talinolol or tertatolol or tilisolol or timolol or toliprolol or xibenolol).mp

33. exp calcium channel blockers/

34. (calcium adj2 (inhibit$ or agonist? or exogenous or blockader?)).tw

35. (calcium channel blockers or amlodipine or amrinone or bencyclane or bepridil or cinnarizine or conotoxins or diltiazem or felodipine or fendiline or flunarizine or gallopamil or isradipine or lidoflazine or magnesium sulfate or mibefradil or nicardipine or nifedipine or nimodipine or nisoldipine or nitrendipine or perhexiline or prenylamine or verapamil or omega-agatoxin iva or omega-conotoxin gvia or omega-conotoxins).mp

36. exp diuretics/

37. (thiazide or furosemide or bumetanide or piretanide or torasemide or azosemide or ethacrynic acid or ticrynafen or trip amide or phenoxybenzoic acid or muzolimine or indacrinone or etozolin or ozolinone or cicletanine or cicletanine or tienilic acid or tizolemide or hydrochlorothiazide or chlorothiazide or buthiazide or bendroflumethiazide or hydroflumethiazide or trichlormethiazide or methylclothiazide or polythiazide or cyclothiazide or cyclopenthiazide or chlorthalidone or metolazone or quinethazone or fenquizone or clorexolone or clop amide or indapamide or diapamide or isodapamide or mefruside or xipamide or diuretic$).mp

38. exp adrenergic alpha antagonists/

39. (alpha adrenergic antagonist? or alpha adrenergic receptor antagonist? or adrenergic alpha antagonist?).tw

40. ((alpha or alpha-adrenergic) adj2 block$).tw

41. (alfuzosin or bunazosin or doxazosin or indoramin or metazosin or neldazosin or prazosin or silodosin or tamsulosin or terazosin or tiodazosin or trimazosin or urapidil or phentolamine or phenoxybenzamine).mp

42. (methyldopa or alphamethyldopa or amodopa or dopamet or dopegyt or dopegit or dopegite or emdopa or hyperpax or hyperpaxa or methylpropionic acid or dopergit or meldopa or methyldopate or medopa or medomet or sembrina or aldomet or aldometil or aldomin or hydopa or methyldihydroxyphenylalanine or methyl dopa or mulfasin or presinol

or presolisin or sedometil or sembrina or taquinil or dihydroxyphenylalanine or methylphenylalanine or methylalanine or alpha methyl dopa).mp.

43. (alazine or alphapress or apdormin or apresolin or apresolina or apresoline or apressin or apressoline or aprezin or clorana or depressan or deselazin or dihydralazine or dihydrallazine or dihydrazinophthalazin or dralzine or hidral or hydralacin or hydralazine or hydralazine or hydralizine or hydrallazin or hydralazine or hydrapres or hydrazinophtalizine or hydrazinophthalazine or hydrolazine or hypatol or hyperphen or hypoftalin or hypophthalin or idralazina or iopress or nepresol or nonpolin or novo-hylazin or plethorit or praeparat or resporidin or solesorin or tetrasoline or travinon).mp

44. exp nitroprusside/

45. aliskiren.mp

46. renin inhibitor$.mp

47. moxonidine.mp

48. (minoxidil or minoxidine).mp. [mp=title, abstract, original title, name of substance word, subject heading word, keyword heading word, protocol supplementary concept word, rare disease supplementary concept word, unique identifier, synonyms]

49. clonidine.mp

50. guanfacine.mp

51. 17 or 18 or 19 or 20 or 21 or 22 or 23 or 24 or 25 or 26 or 27 or 28 or 29 or 30 or 31 or 32 or 33 or 34 or 35 or 36 or 37 or 38 or 39 or 40 or 41 or 42 or 43 or 44 or 45 or 46 or 47 or 48 or 49 or 50

52. 16 or 51

53. randomized controlled trial/

54. randomization/

55. Clinical Trial/

56. (clin$ adj25 trial$).ti,ab

57. ((singl$ or doubl$ or trebl$ or tripl$) adj25 (blind$ or mask$)).ti,ab

58. placebo$.ti,ab

59. random$.ti,ab

60. 53 or 54 or 55 or 56 or 57 or 58 or 59

61. exp animals/ not humans.sh.

62. 60 not 61

63. 5 and 52 and 62

Embase search strategy

1. exp basal ganglia hemorrhage/ or intracranial hemorrhages/ or cerebral hemorrhage/ or intracranial hemorrhage, hypertensive/ or cerebrovascular disorders/

2. ((brain$ or cerebr$ or cerebell$ or intracerebral or intracran$ or parenchymal or intraparenchymal or intraventricular or infratentorial or supratentorial or basal gangli$ or putaminal or putamen or posterior fossa or hemispher$ or pon$ or lentiform$ or brainstem or cortic$ or cortex$ or subcortic$ or subcortex$) adj3 (h? emorrhag$ or h?ematoma$ or bleed$)).tw

3. ((hemorrhag$ or haemorrhag$) adj3 (stroke$ or apoplex$ or cerebral vasc$ or cerebrovasc$ or cva)).tw

4. (ICH or ICHs or PICH or PICHs).tw

5. 1 or 2 or 3 or 4

6. exp blood pressure/

7. exp hypertension/

8. (blood pressure or bloodpressure).tw

9. (hypertension or hypertensive).tw

10. ((bp or blood pressure) adj3 (lowering or reduc$)).tw

11. ((strict$ or target$ or tight$ or intens$ or below) adj3 (blood pressure or systolic or diastolic or bp or level$)).tw

12. ((manage$ or monitor$) adj3 (hypertension or blood pressure)).tw

13. ((intense or intensive or aggressive or accelerated or profound or radical or severe) adj3 ((bp or blood pressure) adj3 (lowering or reduc$ or decreas$ or decrement or dimin$ or declin$))).tw

14. ((standard or normal or ordinary or guideline or guide line or guideline recommend$ or recommend$ or convention$ or usual or established) adj3 ((bp or blood pressure) adj3 (lowering or reduc$ or decreas$ or decrement or dimin$ or declin$))).tw

15. (antihypertensive adj2 (agent$ or drug$ or medicat$)).tw

16. exp antihypertensive agents/

17. exp antihypertensive therapy/

18. 6 or 7 or 8 or 9 or 10 or 11 or 12 or 13 or 14 or 15 or 16 or 17

19. exp glyceryl trinitrate/

20. nitrate.tw

21. glyceryl trinitrate.mp. or GTN.tw

22. nitric Oxide Donors.tw

23. exp peptidyl carboxypeptidase inhibitor/

24. exp ace inhibitor/

25. angiotensin-converting enzyme inhibitor$.mp

26. ace inhibitor$.mp

27. (alacepril or altiopril or benazepril or captopril or ceronapril or cilazapril or delapril or derapril or enalapril or fosinopril or idapril or imidapril or lisinopril or moexipril or moveltipril or pentopril or perindopril or quinapril or ramipril or spirapril or temocapril or trandolapril or zofenopril).mp

28. exp Angiotensin Receptor Antagonists/

29. (angiotensin adj3 (receptor antagon$ or receptor block$)).tw

30. arb?.tw

31. (abitesartan or azilsartan or candesartan or elisartan or embusartan or eprosartan or forasartan or irbesartan or losartan or milfasartan or olmesartan or saprisartan or tasosartan or telmisartan or valsartan or zolasartan).mp

32. exp beta adrenergic receptor blocking agent/

33. (beta adj2 (adrenergic? or antagonist? or block$ or receptor?)).tw

34. (acebutolol or alprenolol or amosulalol or arotinolol or atenolol or befunolol or betaxolol or bevantolol or bisoprolol or bopindolol or bucindolol or bucumolol or bufetolol or bufuralol or bunitrolol or bupranolol or butofilolol or carazolol or carteolol or carvedilol or celiprolol or cetamolol or cloranolol or cyanopindolol or deacetylmetipranolol or dihydroalprenolol or dilevalol or epanolol or esmolol or indenolol or iodocyanopindolol or labetalol or landiolol or levobunolol or mepindolol or metoprolol or metipranolol or moprolol or nadolol or nadoxolol or nebivolol or nifenalol or nipradilol or oxprenolol or penbutolol or pindolol or practolol or

pronethalol or propranolol or proxodolol or sotalol or sulfinalol or talinolol or tertatolol or tilisolol or timolol or toliprolol or xibenolol).mp

35. exp calcium channel blocking agent/

36 (calcium adj2 (inhibit$ or agonist? or exogenous or blockader?)).tw

37. (calcium channel blockers or amlodipine or amrinone or bencyclane or bepridil or cinnarizine or conotoxins or diltiazem or felodipine or fendiline or flunarizine or gallopamil or isradipine or lidoflazine or magnesium sulfate or mibefradil or nicardipine or nifedipine or nimodipine or nisoldipine or nitrendipine or perhexiline or prenylamine or verapamil or omega-agatoxin iva or omegaconotoxin gvia or omega-conotoxins).mp

38. exp diuretic agent/

39. (thiazide or furosemide or bumetanide or piretanide or torasemide or azosemide or ethacrynic acid or ticrynafen or trip amide or phenoxybenzoic acid or muzolimine or indacrinone or etozolin or ozolinone or cicletanine or cicletanine or tienilic acid or tizolemide or hydrochlorothiazide or chlorothiazide or buthiazide or bendroflumethiazide or hydroflumethiazide or trichlormethiazide or methylclothiazide or polythiazide or cyclothiazide or cyclopenthiazide or chlorthalidone or metolazone or quinethazone or fenquizone or clorexolone or clop amide or indapamide or diapamide or isodapamide or mefruside or xipamide or diuretic$).mp

40. exp alpha adrenergic receptor blocking agent/

41. (alpha adrenergic antagonist? or alpha adrenergic receptor antagonist? or adrenergic alpha antagonist?).tw

42. ((alpha or alpha-adrenergic) adj2 block$).tw

43. (alfuzosin or bunazosin or doxazosin or indoramin or metazosin or neldazosin or prazosin or silodosin or tamsulosin or terazosin or tiodazosin or trimazosin or urapidil or phentolamine or phenoxybenzamine).mp

44. (methyldopa or alphamethyldopa or amodopa or dopamet or dopegyt or dopegit or dopegite or emdopa or hyperpax or hyperpaxa or methylpropionic acid or dopergit or meldopa or methyldopate or medopa or medomet or sembrina or aldomet or aldometil or aldomin or hydopa or methyldihydroxyphenylalanine or methyl dopa or mulfasin or presinol or presolisin or sedometil or sembrina or taquinil or dihydroxyphenylalanine or methylphenylalanine or methylalanine or alpha methyl dopa).mp

45. (alazine or alphapress or apdormin or apresolin or apresolina or apresoline or apressin or apressoline or aprezin or clorana or depressan or deselazin or dihydralazine or dihydrallazine or dihydrazinophthalazin or dralzine or hidral or hydralacin or hydralazine or hydralazine or hydralizine or hydrallazin or hydralazine or hydrapres or hydrazinophtalizine or hydrazinophthalazine or hydrolazine or hypatol or hyperphen or hypoftalin or hypophthalin or idralazina or iopress or nepresol or nonpolin or novo-hylazin or plethorit or praeparat or resporidin or solesorin or tetrasoline or travinon).mp

46. exp nitroprusside sodium/

47. aliskiren.mp

48. renin inhibitor$.mp

49. moxonidine.mp

50. (minoxidil or minoxidine).mp. [mp=title, abstract, original title, name of substance word, subject heading word, keyword heading word, protocol supplementary concept word, rare disease supplementary concept word, unique identifier] {Including Related Terms}

51. clonidine.mp

52. guanfacine.mp

53. 19 or 20 or 21 or 22 or 23 or 24 or 25 or 26 or 27 or 28 or 29 or 30 or 31 or 32 or 33 or 34 or 35 or 36 or 37 or 38 or 39 or 40 or 41 or 42 or 43 or 44 or 45 or 46 or 47 or 48 or 49 or 50 or 51 or 52

54. 18 or 53

55. crossover-procedure.sh

56. double-blind procedure.sh

57. single-blind procedure.sh

58. (random$ or factorial$ or crossover$ or cross over$ or cross-over$ or placebo$ or (doubl$ adj blind$) or (singl$ adj blind$) or assign$ or allocat$).tw,ot

59. randomized controlled trial.sh

60. trial.ti

61. controlled clinical trial/

62. 55 or 56 or 57 or 58 or 59 or 60 or 61

63. exp animal/ or exp invertebrate/ or animal.hw. or non human/ or nonhuman/

64. human/ or human cell/ or human tissue/ or normal human/

65. 63 not 64

66. 62 not 65

Cochrane Central Register of Controlled Trials

#1 MeSH descriptor: [Basal Ganglia Hemorrhage] explode all trees

#2 MeSH descriptor: [Intracranial Hemorrhage, Hypertensive] explode all trees

#3 MeSH descriptor: [Cerebral Hemorrhage] explode all trees

#4 MeSH descriptor: [Cerebrovascular Disorders] this term only

#5 (hemorrhag* or haemorrhag*) ti.ab.kw

#6 (stroke* or apoplex* or cerebral vasc*? or cerebrovasc* or cva) ti.ab.kw

#7 #5 and #6

#8 (brain* or cerebr* or cerebell* or intracerebral or intracran* or parenchymal or infratentorial or supratentorial or basal gangli*? or putaminal or putamen or posterior fossa? or pons or pontine or brainstem or lentiform) ti.ab.kw

#9 (haemorrhage* or hemorrhage* or haematoma* or hematoma* or bleed*) ti.ab.kw

#10 #8 and #9

#11 #1 or #2 or #3 or #4 or #7 or #10

#12 ((bp or blood pressure) adj5 (lowering or reduc$)) .tw

#13 ((strict$ or target$ or tight$ or intens$ or below) adj3 (blood pressure or systolic or diastolic or bp or level$)) .tw

#14 (hypertension or hypertensive) .tw

#15 ((manage$ or monitor$) adj3 (hypertension or blood pressure)) .tw

#16 ((intense or intensive or aggressive or accelerated or profound or radical or severe) adj5 ((bp or blood pressure) adj5 (lowering or reduc$ or decreas$ or decrement or dimin$ or declin $))) .tw

#17 ((standard or normal or ordinary or guideline or guide line or guideline recommend$ or recommend$ or convention$ or usual or established) adj5 ((bp or blood pressure) adj5 (lowering or reduc$ or decreas$ or decrement or dimin$ or declin$))) .tw

#18 (antihypertensive adj2 (agent$ or drug$ or medicat$)) .tw

#19 MeSH descriptor: [Antihypertensive Agents] explode all trees

#20 #12 or #13 or #14 or #15 or #16 or #17 or #18 or #19

#21 MeSH descriptor: [Angiotensin-Converting Enzyme Inhibitors] explode all trees

#22 angiotensin-converting enzyme inhibitor$.mp

#23 ace inhibitor$.mp

#24 (alacepril or altiopril or benazepril or captopril or ceronapril or cilazapril or delapril or derapril or enalapril or fosinopril or idapril or imidapril or lisinopril or moexipril or

moveltipril or pentopril or perindopril or quinapril or ramipril or spirapril or temocapril or trandolapril or zofenopril) .mp

#25 MeSH descriptor: [Angiotensin Receptor Antagonists] explode all trees 2059

#26 (angiotensin adj3 (receptor antagon$ or receptor block $)) .tw

#27 arb?.tw

#28 (abitesartan or azilsartan or candesartan or elisartan or embusartan or eprosartan or forasartan or irbesartan or losartan or milfasartan or olmesartan or saprisartan or tasosartan or telmisartan or valsartan or zolasartan) .mp

#29 MeSH descriptor: [Adrenergic beta-Antagonists] explode all trees 4548

#30 (beta adj2 (adrenergic? or antagonist? or block$ or receptor?)) .tw

#31 (acebutolol or alprenolol or amosulalol or arotinolol or atenolol or befunolol or betaxolol or bevantolol or bisoprolol or bopindolol or bucindolol or bucumolol or bufetolol or bufuralol or bunitrolol or bupranolol or butofilolol or carazolol or carteolol or carvedilol or celiprolol or cetamolol or cloranolol or cyanopindolol or deacetylmetipranolol or dihydroalprenolol or dilevalol or epanolol or esmolol or indenolol or iodocyanopindolol or labetalol or landiolol or levobunolol or mepindolol or metoprolol or metipranolol or moprolol or nadolol or nadoxolol or nebivolol or nifenalol or nipradilol or oxprenolol or penbutolol or pindolol or practolol or pronethalol or propranolol or proxodolol or sotalol or sulfinalol or talinolol or tertatolol or tilisolol or timolol or toliprolol or xibenolol) .mp

#32 MeSH descriptor: [Calcium Channel Blockers] explode all trees

#33 (calcium adj2 (inhibit$ or agonist? or exogenous or blockader?)) .tw

#34 (calcium channel blockers or amlodipine or amrinone or bencyclane or bepridil or cinnarizine or conotoxins or diltiazem or felodipine or fendiline or flunarizine or gallopamil or isradipine or lidoflazine or magnesium sulfate or mibefradil or nicardipine or nifedipine or

nimodipine or nisoldipine or nitrendipine or perhexiline or prenylamine or verapamil or omega-agatoxin iva or omegaconotoxin gvia or omega-conotoxins) .mp

#35 MeSH descriptor: [Diuretics] explode all trees

#36 (thiazide or furosemide or bumetanide or piretanide or torasemide or azosemide or ethacrynic acid or ticrynafen or trip amide or phenoxybenzoic acid or muzolimine or indacrinone or etozolin or ozolinone or cicletanine or cicletanine or tienilic acid or tizolemide or hydrochlorothiazide or chlorothiazide or buthiazide or bendroflumethiazide or hydroflumethiazide or trichlormethiazide or methylclothiazide or polythiazide or cyclothiazide or cyclopenthiazide or chlorthalidone or metolazone or quinethazone or fenquizone or clorexolone or clop amide or indapamide or diapamide or isodapamide or mefruside or xipamide or diuretic$) .mp

#37 MeSH descriptor: [Adrenergic alpha-Antagonists] explode all trees

#38 (alpha adrenergic antagonist? or alpha adrenergic receptor

antagonist? or adrenergic alpha antagonist?) .tw

#39 ((alpha or alpha-adrenergic) adj2 block$) .tw

#40 (alfuzosin or bunazosin or doxazosin or indoramin or metazosin or neldazosin or prazosin or silodosin or tamsulosin or terazosin or tiodazosin or trimazosin or urapidil or phentolamine or phenoxybenzamine) .mp

#41 (methyldopa or alphamethyldopa or amodopa or dopamet or dopegyt or dopegit or dopegite or emdopa or hyperpax or hyperpaxa or methylpropionic acid or dopergit or meldopa or methyldopate or medopa or medomet or sembrina or aldomet or aldometil or aldomin or hydopa or methyldihydroxyphenylalanine or methyl dopa or mulfasin or presinol or presolisin or sedometil or sembrina or taquinil or dihydroxyphenylalanine or methylphenylalanine or methylalanine or alpha methyl dopa) .mp

#42 (alazine or alphapress or apdormin or apresolin or apresolina or apresoline or apressin or apressoline or aprezin or clorana or depressan or deselazin or dihydralazine or dihydrallazine or dihydrazinophthalazin or dralzine or hidral or hydralacin or hydralazine or hydralazine or hydralizine or hydrallazin or hydralazine or hydrapres or hydrazinophtalizine or hydrazinophthalazine or hydrolazine or hypatol or hyperphen or hypoftalin or hypophthalin or idralazina or iopress or nepresol or nonpolin or novo-hylazin or plethorit or praeparat or resporidin or solesorin or tetrasoline or travinon) .mp

#43 MeSH descriptor: [Nitroprusside] explode all trees

#44 aliskiren.mp

#45 renin inhibitor$.mp

#46 moxonidine.mp

#47 (minoxidil or minoxidine) .mp

#48 clonidine.mp

#49 guanfacine.mp

#50 MeSH descriptor: [Nitroglycerin] explode all trees

#51 #21 or #22 or #23 or #24 or #25 or #26 or #27 or #28 or #29 or #30 or #31 or #32 or #33 or #34 or #35 or #36 or #37 or #38 or #39 or #40 or #41 or #42 or #43 or #44 or #45 or #46 or #47 or #48 or #49 or #50 17095

#52 #20 or #51 23976

#53 #11 and #52 306
