# Supplementary material for: Lowering blood pressure after acute intracerebral haemorrhage: protocol for a systematic review and meta-analysis using individual patient data from randomised controlled trials participating in the Blood Pressure in Acute Stroke Collaboration (BASC)
Source: BMJ Open. 2019 Jul 16;9(7):e030121. doi: 10.1136/bmjopen-2019-030121 (PMC6661570; doi:10.1136/bmjopen-2019-030121)
Supplement: Supplementary data [file bmjopen-2019-030121supp002.pdf]

SUPPLEMENTARY MATERIAL 2: Variables for extraction

Trial information	**Name of trial/acronym, chief investigator/study group, years conducted, number of participants randomised, number of participants treated (intervention vs. controls), number of participants with primary outcome.**
Demographics	**Age, sex,** ethnicity**, country**.
Medical history [Y/N]	Premorbid disability (mRS), **hypertension**, diabetes mellitus, ischaemic stroke, intracerebral haemorrhage, transient ischaemic attack, ischaemic heart disease including angina and myocardial infarction, congestive cardiac failure, atrial fibrillation, valvular heart disease, peripheral arterial disease, hyperlipidaemia, family history of young stroke, current alcohol excess, smoker (active/previous/never).
Medications at time of admission [Y/N]	**BP lowering therapy**: ACEi, ARB, BB, CCB, diuretic, AB, nitric oxide donor, centrally acting, other<br>**Anticoagulant therapy**: warfarin or non-VKA oral anticoagulant<br>**Antiplatelet therapy**: aspirin, clopidogrel, cilostazol, dipyridamole, other, two or more<br>Lipid lowering therapy: statin, other
Baseline clinical variables	**Systolic BP [mmHg], diastolic BP [mmHg], heart rate [beats per min],** presence of AF on electrocardiogram [Y/N], dysphagia [Y/N], **NIHSS score [/42], GCS score [/15]**, Scandinavian Neurological Stroke Scale [/58], stroke syndrome [TACS/PACS/LACS/POCS].
Baseline blood tests	eGFR [mL/min], creatinine [μmol/L or mg/dL], urea [mmol/L], sodium [mmol/L], potassium [mmol/L] blood glucose [mmol/L or mg/dL], osmolarity [calculated].
Baseline neuroimaging characteristics	**Scan availability [Y/N]**, CT [Y/N], CT-A [Y/N], MRI [Y/N], **ICH volume [mm^3 or mL], ICH location** [lobar or basal ganglia/deep or infratentorial/brainstem/cerebellar], **presence of intraventricular haemorrhage [Y/N]**, IVH volume [mm^3 or mL], CT-A spot sign [Y/N], mass effect [Y/N], perihaematomal oedema volume [mm^3 or mL], small vessel disease [Y/N], brain atrophy [Y/N], evidence of old stroke [Y/N].
BP treatment	**Time from onset to treatment [hours:minutes], treatment group [intensive vs. standard or intervention vs. control],** route of administration [IV/oral/transdermal/sublingual]

	IV antihypertensive agents: BB, CCB, diuretic, AB, nitric oxide donor, nitroprusside, hydralazine, other –name [Y/N]<br><br>Transdermal agents: GTN patch [Y/N]<br><br>Oral antihypertensive agent: ACEi, ARB, BB, CCB, diuretic, AB, central acting, other – name [Y/N]<br><br>Time to achieve target BP [hours:minutes]<br><br>**All trial BP and heart rate measurements within first 168 hours.**
Other management [Y/N]	Intubation, ICU/ITU, surgery [evacuation/ intraventricular drainage/ minimally invasive surgery/ other – name], VTE prophylaxis [heparin/ compression stockings/ intermittent pneumatic compression], mannitol, haemostatic agents, DNAR/withdrawal of active treatment.
Outcomes	**Death: within 90 days [Y/N]**<br><br>**Disability: mRS at day 7 and 90 [0-6],** NIHSS score at days 2, 3 and 7, Barthel index at day 7, 90, 180.<br><br>Quality of life: 5-level EQ5D score at 90 days [/1]<br><br>Cognitive function: MMSE at 90 days [/30]<br><br>**Serious adverse events: all fatal and non-fatal,** renal, VTE, pneumonia**, early neurologic deterioration, symptomatic hypotension requiring corrective therapy**<br><br>Haematoma characteristics on repeat brain imaging: ICH volume [mm^3 or mL], IVH volume [mm^3 or mL], perihaematomal oedema volume [mm^3 or mL]

Bold text indicates core variable

mRS indicates modified Rankin Scale; ACEi, angiotensin-converting enzyme inhibitor; ARB, angiotensin receptor blocker; BB, beta blocker; CCB, calcium channel blocker; AB, alpha blocker; BP, blood pressure; AF, atrial fibrillation; NIHSS, National Institute for Health Stroke Scale; GCS, Glasgow coma scale; TACS, total anterior circulation syndrome; PACS, partial anterior circulation syndrome; LACS, lacunar syndrome; POCS, posterior circulation syndrome; eGFR, estimated glomerular filtration rate; CT, computed tomography; CT-A, computed tomographic angiography; MRI, magnetic resonance imaging; ICH, intracerebral haemorrhage; IV, intravenous; GTN, glyceryl trinitrate; ICU/ITU, intensive care/treatment unit; VTE, venous thromboembolism; DNAR, do not attempt resuscitation, EQ-5D EuroQOL five dimensions health-related quality of life questionnaire
