# Supplementary material for: Lowering blood pressure after acute intracerebral haemorrhage: protocol for a systematic review and meta-analysis using individual patient data from randomised controlled trials participating in the Blood Pressure in Acute Stroke Collaboration (BASC)
Source: BMJ Open. 2019 Jul 16;9(7):e030121. doi: 10.1136/bmjopen-2019-030121 (PMC6661570; doi:10.1136/bmjopen-2019-030121)
Supplement: Supplementary data [file bmjopen-2019-030121supp003.pdf]

SUPPLEMENTARY MATERIAL 3: Table shells

TABLE 1. Baseline characteristics according to treatment group. Data are number (%), mean (standard deviation) or median (interquartile range), chi-squared or Kruskal-Wallis tests will be used to make comparisons.

	Active	Control	P
Age (years)			
Sex, female			
Geographical region			
North America			
Asia			
Europe			
Other			
ICH onset to randomisation (hrs)			
Medical history			
Hypertension			
Diabetes mellitus			
Stroke			
Ischaemic heart disease			
AF (history or ECG finding)			
Smoker			
Medication history			
Antihypertensive			
Antiplatelet			
Anticoagulation			
Clinical			
Systolic BP (mmHg)			
Diastolic BP (mmHg)			
Pulse pressure (mmHg)			
Heart rate (bpm)			

Rate-pressure product[1]	
NIHSS (/42)	
GCS (/15)	
Neuroimaging	
Haematoma volume (ml)	
Haematoma location	
Lobar	
Basal ganglia/deep	
Infratentorial	
Intraventricular haemorrhage	
Perihaematomal oedema	
Absolute (ml)	
Relative[2]	
Pre-stroke characteristics	
Leukoaraiosis	
Atrophy	
Prior stroke lesion	

ICH denotes intracerebral haemorrhage; AF, atrial fibrillation; BP, blood pressure; NIHSS, National Institute for Health stroke scale; GCS, Glasgow come scale

[1]Systolic BP multiplied by heart rate

[2]Perihaematomal oedema to intraparenchymal haemorrhage volume ratio

TABLE 2. Treatment used to lower blood pressure, and other processes of care. Data are number (%). Chi squared tests will be used to make comparisons.

	Treatment	Control	P
Route of administration			
Oral			
Intravenous			
Transdermal			
Sublingual			
BP intervention ^			
Target *			
ACE-I, po/sl §			
ARA, po +			
ß-RA, po/im §§			
CCB, iv ¥			
CCB, po $			
Nitrate, topical #			
Magnesium, iv ʃ			
Other treatments (%)			
Mannitol			
Haemostatic agents			
VTE prophylaxis			
DNAR order/withdrawal of active treatment			
ITU/intubation			
Neurosurgery			

BP denotes blood pressure; ACE-I, angiotensin converting enzyme inhibitor; ARA, angiotensin receptor antagonist; ß-RA, beta receptor antagonist; CCB, calcium channel blocker; VTE, venous thromboembolism; DNAR, do not attempt resuscitation; ITU, intensive treatment unit; po, oral; sl, sublingual; im, intramuscular; iv, intravenous

^ Categorised according to current BASC collaborator trials

*INTERACT1&2, ATACH-II, ICH-ADAPT; § CHHIPS, ACEi arm; + SCAST; §§ CHHIPS, ß-RA arm; ¥ ATACH-II, Uzuner; $ VENUS; # GTN1&2, ENOS, RIGHT; ʃ IMAGES, FAST-Mag

TABLE 3. Effect of BP lowering on outcomes. Data are number (%), mean (standard deviation) or median (interquartile range); mean difference (MD) or odds ratios (OR) with 95% confidence intervals (CI). Generalised linear mixed models for binary, continuous and ordinal outcomes, with random intercept for source trial. Multivariable analyses adjusted for age, sex, severity (NIHSS), systolic BP, ICH volume, presence of IVH and time to randomisation.

	N	Treatment	Control	OR/MD	95% CI	P	OR/MD	95% CI	P
				Unadjusted			Adjusted		
**On treatment outcomes**									
Within 1 hour of randomisation									
Systolic BP (mmHg)									
Mean[1]				MD			MD		
Delta[2]				MD			MD		
Peak[3]				MD			MD		
Standard deviation[4]				MD			MD		
Diastolic BP § (mmHg)				MD			MD		
Pulse pressure § (mmHg)				MD			MD		
Heart rate § (bpm)				MD			MD		
Rate pressure product[5]				MD			MD		
Within 24 hours of randomisation									
Systolic BP (mmHg)									

Mean[1]	MD	MD
Delta[2]	MD	MD
Peak[3]	MD	MD
Standard deviation[4]	MD	MD
Diastolic BP § (mmHg)	MD	MD
Pulse pressure § (mmHg)	MD	MD
Heart rate § (bpm)	MD	MD
Rate pressure product[5]	MD	MD
Neurologic deterioration	OR	OR
Symptomatic hypotension	OR	OR
Within 7 days of randomisation		
Systolic BP (mmHg)		
Mean[1]	MD	MD
Delta[2]	MD	MD
Peak[3]	MD	MD
Standard deviation[4]	MD	MD
Diastolic BP § (mmHg)	MD	MD
Pulse pressure § (mmHg)	MD	MD
Heart rate § (bpm)	MD	MD

Rate pressure product[5]	MD	MD
Neurologic deterioration	OR	OR
Symptomatic hypotension	OR	OR
**Repeat brain imaging**		
Completed within 48 hours	OR	OR
Haematoma volume		
Absolute (ml)	MD	MD
Expansion		
>33%	OR	OR
>6ml	OR	OR
Perihaematomal oedema volume		
Absolute (ml)	MD	MD
Relative[6]	MD	MD
Mass effect	OR	OR
**90-day clinical outcomes**		
Primary outcome (unfavourable shift in ordinal mRS score)	OR	OR
Death (%)	OR	OR

Dichotomised mRS score		
4-6, death and major disability (%)	OR	OR
3-6, death and dependency (%)	OR	OR
Barthel index (/100)	MD	MD
EQ-5D-5L (/25)	MD	MD
MMSE (/30)	MD	MD
Serious adverse events		
Fatal	OR	OR
Non-fatal	OR	OR
Renal	OR	OR
Cardiac	OR	OR
Pneumonia	OR	OR
VTE	OR	OR

BP denotes blood pressure; SD, standard deviation; bpm, beats per minute; mRS, modified Rankin Scale; EQ-5D-5L, European Quality of Life–5 Dimensions 5-level questionnaire; MMSE, mini mental state examination; VTE, venous thromboembolism.

[1]Mean of measures within specified period

[2]Baseline minus mean within specified period

[3]Highest value within specified period

[4]Standard deviation of measures within specified period

[5]Peak systolic BP multiplied by peak heart rate within specified period

§Mean, delta, peak and standard deviation will also be assessed

FIGURE 1. Shift in mRS according to BP lowering vs. control/guideline treatment. Give adjusted odds ratio from generalised linear mixed model with source trial as random effect. Adjusted for age, sex, severity (NIHSS), systolic BP, ICH volume at baseline, IVH at baseline, time to randomisation.

FIGURE 2. Forest plot of shift in mRS by pre-defined subgroups. Same model as figure 1 with interaction tests.

- Age: ≤70, >70 (years)
- Sex
- Region: Asian vs other
- Pre-stroke hypertension: Y/N
- Pre-stroke antithrombotic use: Y/N
- Baseline SBP: ≤140, 140-180, 180-220, >220 (mmHg)
- NIHSS: ≤ 8, 9-15, >15
- Haematoma volume at baseline: ≤ 15, >15 (ml)
- Type of intervention: target vs angiotensin inhibitor vs CCB vs NO donor vs magnesium/other
- Time from stroke to randomisation: <2, 2-6, 6-48, >48 (hours)
- Type of trial: pre-hospital vs hospital, ICH only vs mixed

FIGURE 3. Kaplan-Meier curve for death with comparison of active and control treatment groups. Model adjusted for age, sex, severity (NIHSS), systolic BP, ICH volume at baseline, IVH at baseline, time to randomisation and source trial.

FIGURE 4. Forest plot for end-of-trial death by pre-defined subgroups. Same model as figure 1 with interaction tests.

- Age: ≤70, >70 (years)
- Sex
- Region: Asian vs other
- Pre-stroke hypertension: Y/N
- Pre-stroke antithrombotic use: Y/N
- Baseline SBP: ≤140, 140-180, 180-220, >220 (mmHg)
- NIHSS: ≤ 8, 9-15, >15
- Haematoma volume at baseline: ≤ 15, >15 (ml)
- Type of intervention: target vs angiotensin inhibitor vs CCB vs NO donor vs magnesium/other
- Time from stroke to randomisation: <2, 2-6, 6-48, >48 (hours)
- Type of trial: pre-hospital vs hospital, ICH only vs mixed

FIGURE 5. Individual participant data regression: relationship between randomised treatment group and odds ratio for unfavourable shift in scores on the modified Rankin Scale at end of trial and (i) time to randomisation; (ii) baseline systolic blood pressure; and (iii) baseline haematoma volume. Model (i) is adjusted for age, sex, severity, type of treatment, and source trial; models (ii) and (iii) are adjusted for the same plus time to randomisation.
