# Supplementary material for: Lowering blood pressure after acute intracerebral haemorrhage: protocol for a systematic review and meta-analysis using individual patient data from randomised controlled trials participating in the Blood Pressure in Acute Stroke Collaboration (BASC)
Source: BMJ Open. 2019 Jul 16;9(7):e030121. doi: 10.1136/bmjopen-2019-030121 (PMC6661570; doi:10.1136/bmjopen-2019-030121)
Supplement: Author's manuscript [file bmjopen-2019-030121.draft_revisions.pdf]

BMJ Open is committed to open peer review. As part of this commitment we make the peer review history of every article we publish publicly available.

When an article is published we post the peer reviewers' comments and the authors' responses online. We also post the versions of the paper that were used during peer review. These are the versions that the peer review comments apply to.

The versions of the paper that follow are the versions that were submitted during the peer review process. They are not the versions of record or the final published versions. They should not be cited or distributed as the published version of this manuscript.

BMJ Open is an open access journal and the full, final, typeset and author-corrected version of record of the manuscript is available on our site with no access controls, subscription charges or pay-per-view fees (http://bmjopen.bmj.com).

If you have any questions on BMJ Open's open peer review process please email info.bmjopen@bmj.com

BMJ Open

Lowering blood pressure after acute intracerebral haemorrhage: protocol for a systematic review and meta-analysis using individual patient data from randomised controlled trials participating in the Blood pressure in Acute Stroke Collaboration (BASC)

Journal:	*BMJ Open*
Manuscript ID	bmjopen-2019-030121
Article Type:	Protocol
Date Submitted by the Author:	28-Feb-2019

Complete List of Authors:	Moullaali, Tom; University of Edinburgh, Centre for Clinical Brain Sciences; George Institute for Global Health<br>Wang, Xia; George Institute for Global Health<br>Woodhouse, Lisa; University of Nottingham, Stroke Trials Unit<br>Law, Zhe; University of Nottingham, Stroke Trials Unit; National University of Malaysia<br>Delcourt, Candice; George Institute for Global Health; Royal Prince Alfred Hospital<br>Sprigg, Nicola; University of Nottingham, Stroke Trials Unit; Nottingham University Hospitals NHS Trust, Stroke<br>Krishnan, Kailash; University of Nottingham, Stroke Trials Unit; Nottingham University Hospitals NHS Trust, Stroke<br>Robinson, Thomas; University of Leicester, Department of Cardiovascular Sciences and NIHR Leicester Biomedical Research Centre<br>Wardlaw, Joanna; University of Edinburgh, Centre for Clinical Brain Sciences<br>Salman, Rustam; University of Edinburgh, Centre for Clinical Brain Sciences<br>Berge, Eivind; Oslo University Hospital, Department of Internal Medicine<br>Sandset, Else; Oslo University Hospital, Neurology Department; Norwegian Air Ambulance Foundation, Research and Development<br>Anderson, Craig; George Institute for Global Health; Royal Prince Alfred Hospital, Neurology Department<br>Bath, Philip; University of Nottingham, Stroke Trials Unit; Nottingham University Hospitals NHS Trust, Stroke

Keywords:	Stroke < NEUROLOGY, intracerebral haemorrhage, blood pressure lowering, individual participant data meta-analysis

SCHOLARONE™
Manuscripts

Lowering blood pressure after acute intracerebral haemorrhage: protocol for a systematic review and meta-analysis using individual patient data from randomised controlled trials participating in the Blood pressure in Acute Stroke Collaboration (BASC)

Tom J Moullaali†,[1,2] Xia Wang†,[1] Lisa J Woodhouse,[3] Zhe Kang Law,[3,4,5] Candice Delcourt,[1,6] Nikola Sprigg,[3,4] Kailash Krishnan,[3,4] Thompson G Robinson,[7] Joanna Wardlaw,[2] Rustam Al-Shahi Salman,[2] Eivind Berge,[8] Else Charlotte Sandset,[9,10] Craig S Anderson,[1,6,11] Philip M Bath.[3,4] for the BASC Investigators*

[1]The George Institute for Global Health, Faculty of Medicine, University of New South Wales, NSW, Australia

[2]Centre for Clinical Brain Sciences, University of Edinburgh, Edinburgh, UK

[3]Stroke Trials Unit, Division of Clinical Neuroscience, University of Nottingham, City Hospital Campus, Nottingham, UK

[4]Stroke, Nottingham University Hospitals NHS Trust, Nottingham, UK

[5]National University of Malaysia, Kuala Lumpur, Malaysia

[6]Neurology Department, Royal Prince Alfred Hospital, The University of Sydney, Sydney, NSW, Australia

[7]University of Leicester, Department of Cardiovascular Sciences and NIHR Leicester Biomedical Research Centre, Leicester, UK

[8]Department of Internal Medicine, Oslo University Hospital, Oslo, Norway

[9]Department of Neurology, Oslo University Hospital, Postboks 4956 Nydalen 0424 Oslo, Norway

[10]Research and Development Department, The Norwegian Air Ambulance Foundation, Oslo, Norway

For peer review only

For peer review only - http://bmjopen.bmj.com/site/about/guidelines.xhtml

[11]The George Institute China at Peking University Health Science Center, Beijing, PR China

†Co-first authors

Corresponding Author:

Professor Craig S Anderson

The George Institute for Global Health

PO Box M201, Missenden Road, NSW 2050, AUSTRALIA

T: +61-2-9993-4500; F: +61-2-9993-4502

canderson@georgeinstitute.org.au

Email addresses for writing committee:
tom.moullaali@ed.ac.uk
xwang@georgeinstitute.org.au
l.woodhouse@nottingham.ac.uk
zhe.law@nottingham.ac.uk
cdelcourt@georgeinstitute.org.au
nikola.sprigg@nottingham.ac.uk
kailash.krishnan@nottingham.ac.uk
tgr2@leicester.ac.uk
joanna.wardlaw@ed.ac.uk
rustam.al-shahi@ed.ac.uk
eivind.berge@medisin.uio.no
else@sandset.net
philip.bath@nottingham.ac.uk

Word count body: 2740

Tables: 0

Supplementary materials: 3

For peer review only - http://bmjopen.bmj.com/site/about/guidelines.xhtml

Figures: 0

Key words: acute stroke; intracerebral haemorrhage; blood pressure lowering; hypertension;

For peer review only

For peer review only - http://bmjopen.bmj.com/site/about/guidelines.xhtml

Abstract

Introduction

Conflicting results from multiple randomised trials indicate that the methods and effects of blood pressure (BP) reduction after acute intracerebral haemorrhage (ICH) are complex. The Blood pressure in Acute Stroke Collaboration (BASC) is an international collaboration which aims to determine the optimal management of BP after acute stroke including ICH.

Methods and analysis

A systematic review will be undertaken according to the Preferred Reporting Items for Systematic review and Meta-Analysis of Individual Participant Data (PRISMA-IPD) guideline. Randomised controlled trials of blood pressure management in adults with acute spontaneous (non-traumatic) ICH enrolled within the first 7 days of symptom onset will be included. Authors of studies that meet the inclusion criteria will be invited to share their IPD. The primary outcome will be functional outcome according to the modified Rankin Scale. Safety outcomes will be early neurologic deterioration, symptomatic hypotension and serious adverse events. Secondary outcomes will include death, and neuroradiological and haemodynamic variables. Meta-analyses of pooled IPD using the intention-to-treat dataset of included trials with a one-stage approach, including meta-regression and subgroup analyses to assess modification of the effects of BP lowering by time to treatment, treatment strategy, and patient demographic, clinical and pre-stroke neuroradiological characteristics.

Ethics and dissemination

No new patient data will be collected nor is there any deviation from the original purposes of each study where ethical approvals were granted, therefore further ethical approval is not required. Results will be reported in international peer-reviewed journals.

For peer review only

Article summary

Strengths and limitations of this study:

- The Blood pressure in Acute Stroke Collaboration (BASC) is an international collaboration with the prospective aim of pooling individual participant data (IPD) from randomised controlled trials (RCTs) of blood pressure (BP) management in acute stroke, including intracerebral haemorrhage (ICH).
- Meta-analysis of IPD is regarded as the gold standard for synthesising evidence from multiple RCTs.
- This study aims to collect sufficient data to determine the optimal management of BP in acute ICH and will facilitate comparisons across subgroups according to patient characteristics, time to treatment and treatment strategy.
- This study will identify associations of treatment-related haemodynamic parameters which will inform future research.

Introduction

Rationale

Acute treatments proven to alter the prognosis of stroke due to spontaneous (non-traumatic) intracerebral haemorrhage (ICH) are limited. A 2014 Cochrane review that appraised the totality of evidence for blood pressure (BP) lowering within one week of acute stroke concluded that the supporting evidence was insufficient to make clear recommendations about this intervention, and that the findings were similar for ischaemic stroke and ICH.[1] In regard to ICH, several randomised controlled trials (RCTs) have focussed on early intensive BP reduction and included participants with mild to moderate acute ICH in the hospital setting.[2–4] Other trials commenced BP lowering in the pre-hospital period before diagnostic brain imaging, and by their nature included a mixture of ischaemic stroke and ICH patients, including more severe cases of ICH.[5–7] Data from ICH cases are also available from RCTs which used longer inclusion windows (up to 48 hours).[8–12] Furthermore, various agents and BP targets have been tested, and few trials considered prognostic implications of pre-stroke neuroimaging characteristics. Therefore,

For peer review only - http://bmjopen.bmj.com/site/about/guidelines.xhtml

a substantial and varied body of evidence about BP lowering and outcome after acute ICH is available, but much of it is conflicting.[13]

The Blood pressure in Acute Stroke Collaboration (BASC) is an international collaboration which aims to prospectively pool individual participant data (IPD) from RCTs of BP control after acute stroke, including stroke due to ICH.[14,15] IPD meta-analysis is considered the gold-standard for synthesising evidence from RCTs[16] and in this context, will facilitate multivariable analyses of treatment effects according to time to treatment, treatment strategy used, and baseline characteristics, adjusted for confounding factors.

Primary objective

Determine the effect of BP lowering on clinical outcomes in patients with acute ICH and elevated BP.

Secondary objectives

i) Determine the effect of BP lowering according to baseline patient clinical and neuroradiological characteristics, BP lowering strategy, and timing of the intervention.

ii) Determine the effect of BP lowering on radiological outcomes.

iii) Where sufficient data are available, report associations of on-treatment haemodynamic parameters, adjusted for known confounders.

Methods and analysis

This systematic review and IPD meta-analysis will be performed in accordance with the recommendations made by the methods group of the Cochrane Collaboration (http://ipdmamg.cochrane.org/resources) and the UK Medical Research Council Network of Hubs for Trials Methodology Research.[17] The protocol has been developed in accordance with the Preferred Reporting Items for Systematic reviews and Meta-Analyses of IPD (PRISMA-IPD) checklist,[18] and has been registered with the international prospective register of systematic reviews, PROSPERO.[19]

Eligibility criteria

Study designs

For peer review only - http://bmjopen.bmj.com/site/about/guidelines.xhtml

RCTs of BP reduction during the acute phase (<7 days) of stroke will be included.

Participants

Adults (>18 years) with spontaneous ICH will be included. For trials including both ICH and ischaemic stroke, we will include only patients with ICH. Participants with secondary ICH (e.g. due to trauma, tumour or vascular malformation) will be excluded. Uncertainty about an individual trial's eligibility criteria will be clarified with the appropriate investigator.

Interventions

Trials which involve interventions that lower BP including oral, sublingual, transdermal and intravenous agents, and single or combination therapy, will be considered.

Comparators

Control groups will typically be managed with placebo or according to contemporaneous guideline recommendations.

Outcomes

Trials that report data on death and functional outcome using the seven-level modified Rankin Scale (mRS, where scores range from 0 = no symptoms to 5 = severe disability, and 6 = death) at 90 days will be included. Where outcome data are not reported this way, the nearest time-point or suitable surrogate will be considered. Data regarding other outcomes (survival, quality of life, cognitive function), serious adverse events (SAEs), haematoma characteristics (growth, final volume, mass effect, oedema), and haemodynamic measures will be sought.

Setting

There will be no restrictions on the type of setting.

Language

There will be no language restriction.

Information sources

For peer review only

Trials will be sought using electronic searches of Cochrane Central Register of Controlled Trials, EMBASE, and MEDLINE, and in the reference lists of published systematic reviews and *ad hoc* reviews. Ongoing or unpublished trials will be identified using ClinicalTrials.gov and the World Health Organisation's International Clinical Trials Registry Platform (ICTRP).

Search strategy

Searches of the above bibliographic databases will be conducted using a combination of search terms relevant to the proposed study (supplementary material 1). Databases will be searched from inception to the present. The search strategy will be enhanced through searches of the existing BASC reviews, the authors' publication databases, and reference lists in identified articles.

Selection process

All titles and abstracts will be screened against the eligibility criteria by TJM and ZKL. Any disagreements will be settled by an adjudicator, PMB. The principal investigators of eligible studies will be invited to participate in the collaboration.

Data management

Data sharing

Investigators of eligible studies will receive a written invitation to share their IPD. To ensure transparency, collaborators sharing data with BASC will be asked to sign a data transfer agreement for the predefined and appropriate use of their data according to this protocol.

Data checking

Initial internal analyses will compare data from each trial with their published results to ensure that data are complete and transferred without error: this will include checking the primary outcome, and all baseline variables or secondary outcomes relevant to the proposed analyses. The integrity of all data will be checked and any queries resolved with individual trial investigators.

Data merging

For peer review only - http://bmjopen.bmj.com/site/about/guidelines.xhtml

Datasets obtained from collaborating studies will be combined to form a new master dataset, which will include a variable to indicate the original study.

Confidentiality, data storage and handling

Each collaborator will de-identify their dataset to ensure no patient identifiable information is transferred. Data will be shared electronically, stored on password-protected, encrypted hard disks in a locked room, with daily backup facilitating disaster recovery.[15] Data will not be shared with anyone outside the collaborating group.

Data items

Variables that will be requested from participating investigators are listed in supplementary material 2, and can be outlined as follows:

- Trial information
- Demographics
- Medical history
- Medications at time of admission
- Baseline clinical variables
- Baseline neuroimaging characteristics of the acute ICH and pre-stroke features (leukoaraiosis, atrophy, prior stroke lesions)
- BP treatment and all trial BP data
- Clinical and radiological outcome data

Study outcomes

Primary outcome

The preferred primary effect variable will be functional outcome defined by the ordinal distribution of mRS scores at the end of trial follow-up (usually 3 months).

Secondary outcomes

(i) Death and dependency (3-6 on the mRS); (ii) death or severe disability (4-6 on the mRS) (iii) all-cause death; (iv) health-related quality of life (mobility, self-care, usual

For peer review only - http://bmjopen.bmj.com/site/about/guidelines.xhtml

activities, pain or discomfort, and anxiety or depression), as assessed with the use of the European Quality of Life–5 Dimensions (EQ-5D) questionnaire.

Safety outcomes

(i) Early neurologic deterioration (as defined by each individual trial); (ii) symptomatic hypotension (as defined by each individual trial); (iii) other SAEs, as defined by trial, to include fatal, non-fatal and treatment-related SAEs (including renal SAEs).

Radiological outcomes

(i) Haematoma growth, both absolute and proportional, will be studied where these data were collected by individual trials, and is based on central measurements from semi-automated planimetric[20] or ABC/2[21] methods; (ii) other imaging outcomes (e.g. perihaematomal oedema) will be included in supplemental analyses.

Subgroup analyses

Where adequate data are available, heterogeneity in the effect of BP lowering on outcomes will be assessed in the following subgroups:

- Baseline characteristics: demographic (age, sex, region), clinical (stroke severity, baseline systolic BP, pre-stroke antihypertensive drug use, pre-stroke antithrombotic use), and radiological parameters (ICH volume, pre-stroke characteristics).
- BP lowering strategy: intervention/class-based treatment, BP target-based treatment.
- Timing of intervention: <2 hours, 2-6 hours, 6-48 hours, >48 hours after onset of ICH.
- Type of trial: pre-hospital vs. hospital, ICH only vs. mixed.

Associations of calculated BP parameters and primary outcome

Once all data have been pooled, an assessment of the new dataset will be made with a view to use trial BP measures to calculate important BP lowering haemodynamic parameters, a number of which have been associated with outcome after ICH.[22–25]

For peer review only - http://bmjopen.bmj.com/site/about/guidelines.xhtml

The aim is to determine the prognostic significance of these calculated variables, adjusted for all known confounders.

Risk of bias in individual studies

Two investigators (TJM and ZKL) will assess each included study for bias using the Cochrane Collaboration tool (http://methods.cochrane.org/bias/assessing-risk-bias-included-studies). A judgement of bias will be made according to 6 domains: selection, performance, detection, attrition, reporting and 'other' biases. Disagreements will be resolved with discussion, or by involving PMB if necessary. Any uncertainties about trial design, conduct or analysis methods will be clarified with the individual trial's investigators.

Statistical analyses

Analyses will be performed using the intention-to-treat dataset from each trial and a one-stage approach will be used. Patients without available data, or where the above procedure for missing data cannot be applied, will be excluded from the analyses.

Supplementary material 3 contains table and figure shells that will be used to present our findings. In summary, descriptive analyses will be undertaken in order to identify key similarities and differences between trials, therefore providing context for interpretation of between-trial differences in outcomes. Data will be described as mean (standard deviation) or median (interquartile range) for continuous data, or frequency (percentage) for categorical data, and chi-squared or Kruskal-Wallis tests will be used to make comparisons.

Generalised linear mixed models will be used with covariates (age, sex, ICH severity, time from onset to randomisation), and the source trial added as a random intercept to account for clustering. For completeness, analyses will also be performed unadjusted. Results from binary and ordinal analyses (e.g. death, dichotomised outcomes of mRS scale, neurologic deterioration, serious adverse events, full-scale mRS) will be presented as odds ratios (OR) with 95% confidence intervals (CI). The proportional odds assumption will be checked before ordinal analyses of outcomes on the mRS are undertaken. If the proportional odds assumption is not met, various standard binary cut-

For peer review only - http://bmjopen.bmj.com/site/about/guidelines.xhtml

points for poor outcome on the mRS will be used. Continuous or pseudo-continuous outcome analysis results will be presented as mean difference (MD) with 95% CI. For time-to-event outcomes, a Cox proportional hazards model will be used to determine hazard ratios (HR) with the source trial added as a random effect, and the assumption of proportional hazards will be tested.

Additional analyses will be performed in pre-specified subgroups, with an interaction term in models to test heterogeneity.

Sensitivity analyses will be conducted to test the robustness of our results: (1) aggregate data will be included in a sensitivity analysis to address the issue of data availability bias where IPD cannot be obtained;[26] and (2) a sensitivity analyses will be conducted with and without studies deemed to have high risk of bias.

An assessment of heterogeneity will be performed before data pooling using the Cochrane Q statistic and I^2 statistic.

An assessment of publication bias will be made by visual inspection for funnel plot asymmetry (with and without studies where IPD is obtained) and with Egger's regression test. The Grades of Recommendation, Assessment, Development, and Evaluation (GRADE)[27] will be used to evaluate the quality of the synthesised evidence.

Ethics and dissemination

Ethical approval for the original studies was sought and is documented elsewhere. No new patient data will be collected nor is there any deviation from the original purposes of each study, therefore further ethical approval is not required. Results from this study will be published in international peer-reviewed journals. All publications from this work will be in name of the BASC Investigators.

Discussion

The proposed study will use pooled IPD from RCTs to address the role of BP management in the acute phase of ICH. Conflicting results from multiple clinical trials indicate that BP reduction is not a straightforward question of whether to treat or not. Rather, the problem is complex and needs to take account of patient characteristics,

For peer review only - http://bmjopen.bmj.com/site/about/guidelines.xhtml

physiological parameters including baseline BP, stroke type, the timing of treatment, strategy and class of antihypertensive agent, route of administration, and dose or target BP.

Elevated baseline systolic BP is associated with haematoma expansion,[28] perihaematomal oedema formation,[29] and increased case fatality.[30] There is evidence from some studies that intensive lowering of BP reduces haematoma enlargement,[31] is safe and tolerable,[2,3] and does not alter cerebral blood flow.[32] Current guidelines recommend lowering BP early in the course after ICH and that targeting a goal of systolic BP <140 mmHg is probably safe in patients presenting with an SBP of 150 to 220 mm Hg.[33,34] Nevertheless, other studies reported no effect of BP reduction on haematoma enlargement,[3,4] and the neutral findings of the ATACH-II trial[4] emphasise that all published data need to be appraised together.

Although guidelines recognise the need for very early treatment, none address the role of pre-hospital BP reduction. Further evidence is also required to confirm associations between key haemodynamic variables, such as BP variability,[22] with poor outcome. Furthermore, the influence of patient characteristics that are present prior to ICH on BP lowering interventions is unknown, although these same variables are known to worsen long-term recovery; these include having a prior stroke, white matter lesions or brain atrophy. The proposed investigation aims to provide definitive evidence on these important issues.

Acknowledgements

The protocol paper is written on behalf of the Blood pressure in Acute Stroke Collaboration (BASC, convener PMB). BASC-ICH comprises:

ATACH-II: Adnan I Qureshi, Yuko Palesch

CHHIPS: John Potter

ENOS: Philip M Bath, Nikola Sprigg, Joanna M Wardlaw

FAST-Mag: Jeff Saver, Nerses Sanossian

GTN-1/2/3, RIGHT: Philip M Bath

For peer review only - http://bmjopen.bmj.com/site/about/guidelines.xhtml

ICH-ADAPT: Ken Butcher

IMAGES: Kennedy R Lees, Keith W Muir

INTERACT: Craig S Anderson

INTERACT2: Craig S Anderson, Hisatomi Arima

PILFAST: Gary Ford

RIGHT-2: Philip M Bath, Nikola Sprigg, Joanna M Wardlaw

SCAST: Eivind Berge, Else C Sandset

VENUS: Janneke Horn

Author contributions

PMB, TJM, ECS and XW were responsible for first draft of the protocol. XW and LJW were responsible for statistical oversight. All authors were are responsible for study conception and the current protocol.

Funding statement

BASC has not received specific grants from any funding agency in the public, commercial, or not-for-profit sectors. Potential trials for inclusion have reported their own funding. TJM is a British Heart Foundation clinical research training fellow. PMB is a Stroke Association Professor of Stroke Medicine. PMB and TGR are NIHR Senior Investigators. CSA is a Senior Research Fellow of the National Health and Medical Research Council (NHMRC) of Australia.

Competing interests statement

None declared

Data statement

BASC is performing protocol-driven IPD meta-analyses of trial data shared by investigators. It is not a data repository and requests for the source data should be made to the relevant chief investigator.

For peer review only - http://bmjopen.bmj.com/site/about/guidelines.xhtml

For peer review only

References

1 Bath PMW, Krishnan K. Interventions for deliberately altering blood pressure in acute stroke. *Cochrane database Syst Rev* 2014;**10**:CD000039.

2 Anderson CS, Huang Y, Wang JG, *et al.* Intensive blood pressure reduction in acute cerebral haemorrhage trial (INTERACT): a randomised pilot trial. *Lancet Neurol* 2008;**7**:391–9.

3 Anderson C, Heeley E, Huang Y, *et al.* Rapid blood-pressure lowering in patients with acute intracerebral hemorrhage. *N Engl J Med* 2013;**368**:2355–65.

4 Qureshi AI, Palesch YY, Barsan WG, *et al.* Intensive blood pressure lowering in patients with acute cerebral hemorrhage. *N Engl J Med* 2016;**375**:1033–43.

5 Saver JL, Starkman S, Eckstein M, *et al.* Prehospital use of magnesium sulfate as neuroprotection in acute Stroke. *N Engl J Med* 2015;**372**:528–36.

6 Shaw L, Price C, McLure S, *et al.* Paramedic initiated lisinopril for acute stroke treatment (PIL-FAST): Study protocol for a pilot randomised controlled trial. *Trials* 2011;**12**:152.

7 Ankolekar S, Fuller M, Cross I, *et al.* Feasibility of an ambulance-based stroke trial, and safety of glyceryl trinitrate in ultra-acute stroke: The rapid intervention with glyceryl trinitrate in hypertensive stroke trial. *Stroke* 2013;**44**:3120–8.

8 Sandset EC, Bath PMW, Boysen G, *et al.* The angiotensin-receptor blocker candesartan for treatment of acute stroke (SCAST): A randomised, placebo-controlled, double-blind trial. *Lancet* 2011;**377**:741–50.

9 Bath PMW, Woodhouse L, Scutt P, *et al.* Efficacy of nitric oxide, with or without continuing antihypertensive treatment, for management of high blood pressure in acute stroke (ENOS): a partial-factorial randomised controlled trial. *Lancet* 2015;**385**:617–28.

10 Potter JF, Robinson TG, Ford GA, *et al.* Controlling hypertension and hypotension immediately post-stroke (CHHIPS): a randomised, placebo-controlled, double-blind pilot trial. *Lancet Neurol* 2009;**8**:48–56.

For peer review only - http://bmjopen.bmj.com/site/about/guidelines.xhtml

11 Robinson TG, Potter JF, Ford GA, *et al.* Effects of antihypertensive treatment after acute stroke in the continue or stop post-stroke antihypertensives collaborative study (COSSACS): a prospective, randomised, open, blinded-endpoint trial. *Lancet Neurol* 2010;**9**:767–75.

12 Koch S, Romano JG, Forteza AM, *et al.* Rapid blood pressure reduction in acute intracerebral hemorrhage: Feasibility and safety. *Neurocrit Care* 2008;**8**:316–21.

13 Bath PM, Appleton JP, Krishnan K, *et al*. Blood pressure in acute stroke: to treat or not to treat: that is still the question. *Stroke* 2018;**49**:1784–90.

14 Bath FJ, Bath PMW. What is the correct management of blood pressure in acute stroke? The blood pressure in acute stroke collaboration. *Cerebrovasc Dis* 1997;**7**:205–13.

15 Sandset EC, Sanossian N, Woodhouse LJ, *et al.* Protocol for a prospective collaborative systematic review and meta-analysis of individual patient data from randomized controlled trials of vasoactive drugs in acute stroke: the blood pressure in acute stroke collaboration, stage-3. *Int J Stroke* 2018; 1:1747493018772733.

16 Clarke MJ, Stewart LA. Systematic Reviews: Obtaining data from randomised controlled trials: how much do we need for reliable and informative meta-analyses? *BMJ* 1994;**309**:1007.

17 Tierney JF, Vale C, Riley R, *et al.* Individual participant data (IPD) meta-analyses of randomised controlled trials: guidance on their use. *PLoS Med* 2015;**12**:e1001855.

18 Stewart LA, Clarke M, Rovers M, *et al.* Preferred reporting items for systematic review and meta-analyses of individual participant data: the PRISMA-IPD statement. *JAMA* 2015;**313**:1657–65.

19 Booth A, Clarke M, Dooley G, *et al.* The nuts and bolts of PROSPERO: An international prospective register of systematic reviews. *Syst Rev* 2012;**1**.

20 Bardera A, Boada I, Feixas M, *et al.* Semi-automated method for brain hematoma and edema quantification using computed tomography. *Comput Med Imaging Graph* 2009;**33**:304–11.

For peer review only - http://bmjopen.bmj.com/site/about/guidelines.xhtml

21 Kothari RU, Brott T, Broderick JP, *et al.* The ABCs of measuring intracerebral hemorrhage volumes. *Stroke* 1996;**27**:1304–5.

22 Manning L, Hirakawa Y, Arima H, *et al.* Blood pressure variability and outcome after acute intracerebral haemorrhage: A post-hoc analysis of INTERACT2, a randomised controlled trial. *Lancet Neurol* 2014;**13**:364–73.

23 Wang X, Arima H, Heeley E, *et al.* Magnitude of blood pressure reduction and clinical outcomes in acute intracerebral hemorrhage: Intensive blood pressure reduction in acute cerebral hemorrhage trial study. *Hypertension* 2015;**65**:1026–32.

24 Carcel C, Wang X, Sato S, *et al.* Degree and timing of intensive blood pressure lowering on hematoma growth in intracerebral hemorrhage: intensive blood pressure reduction in acute cerebral hemorrhage trial-2 results. *Stroke* 2016;**47**:1651–3.

25 Arima H, Heeley E, Delcourt C, *et al.* Optimal achieved blood pressure in acute intracerebral hemorrhage: INTERACT2. *Neurology* 2015;**84**:464–71.

26 Ahmed I, Sutton AJ, Riley RD. Assessment of publication bias, selection bias, and unavailable data in meta-analyses using individual participant data: A database survey. *BMJ* 2012;**344**.

27 Iorio A, Spencer FA, Falavigna M, *et al.* Use of GRADE for assessment of evidence about prognosis: rating confidence in estimates of event rates in broad categories of patients. *BMJ* 2015;**350**.

28 Kazui S, Minematsu K, Yamamoto H, *et al.* Predisposing factors to enlargement of spontaneous intracerebral hematoma. *Stroke* 1997;**28**:2370–5.

29 Vemmos KN, Tsivgoulis G, Spengos K, *et al.* Association between 24-h blood pressure monitoring variables and brain oedema in patients with hyperacute stroke. *J Hypertens* 2003;**21**:2167–73.

30 Zhang Y, Reilly KH, Tong W, *et al.* Blood pressure and clinical outcome among patients with acute stroke in inner Mongolia, China. *J Hypertens* 2008;**26**:1446–52.

For peer review only

31 Anderson CS, Huang Y, Arima H, *et al.* Effects of early intensive blood pressure-lowering treatment on the growth of hematoma and perihematomal edema in acute intracerebral hemorrhage: the intensive blood pressure reduction in acute cerebral haemorrhage trial (INTERACT). *Stroke* 2010;**41**:307–12.

32 Butcher K, Jeerakathil T, Emery D, *et al.* The intracerebral haemorrhage acutely decreasing arterial pressure trial: ICH ADAPT. *Int J Stroke* 2010;**5**:227–33.

33 Steiner T, Al-Shahi Salman R, Beer R, *et al.* European Stroke Organisation (ESO) guidelines for the management of spontaneous intracerebral hemorrhage. *Int J Stroke* 2014;**9**:840–55.

34 Hemphill JC, Greenberg SM, Anderson CS, *et al.* Guidelines for the management of spontaneous intracerebral hemorrhage. *Stroke* 2015;**46**:2032–60.

For peer review only

For peer review only - http://bmjopen.bmj.com/site/about/guidelines.xhtml

SUPPLEMENTARY MATERIAL 1: Search Strategy

Medline search strategy

1. exp basal ganglia hemorrhage/ or intracranial hemorrhages/ or cerebral hemorrhage/ or intracranial hemorrhage, hypertensive/ or cerebrovascular disorders/

2. ((brain$ or cerebr$ or cerebell$ or intracerebral or intracran$ or parenchymal or intraparenchymal or intraventricular or infratentorial or supratentorial or basal gangli$ or putaminal or putamen or posterior fossa or hemispher$ or pon$ or lentiform$ or brainstem or cortic$ or cortex$ or subcortic$ or subcortex$) adj5 (h?emorrhag$ or h?ematoma$ or bleed$)).tw

3. ((hemorrhag$ or haemorrhag$) adj6 (stroke$ or apoplex$ or cerebral vasc$ or cerebrovasc$ or cva)).tw

4. (ICH or ICHs or PICH or PICHs).tw

5. 1 or 2 or 3 or 4

6. exp blood pressure/

7. exp hypertension/

8. (blood pressure or bloodpressure).tw

9. ((bp or blood pressure) adj5 (lowering or reduc$)).tw

10. ((strict$ or target$ or tight$ or intens$ or below) adj3 (blood pressure or systolic or diastolic or bp or level$)).tw

11. (hypertension or hypertensive).tw

12. ((manage$ or monitor$) adj3 (hypertension or blood pressure)).tw

13. ((intense or intensive or aggressive or accelerated or profound or radical or severe) adj5 ((bp or blood pressure) adj5 (lowering or reduc$ or decreas$ or decrement or dimin$ or declin$))).tw

14. ((standard or normal or ordinary or guideline or guide line or guideline recommend$ or recommend$ or convention$ or usual or established) adj5 ((bp or blood pressure) adj5 (lowering or reduc$ or decreas$ or decrement or dimin$ or declin$))).tw

15. (antihypertensive adj2 (agent$ or drug$ or medicat$)).tw

For peer review only - http://bmjopen.bmj.com/site/about/guidelines.xhtml

For peer review only

16. 6 or 7 or 8 or 9 or 10 or 11 or 12 or 13 or 14 or 15

17. exp nitroglycerin/

18. nitrate.tw

19. glyceryl trinitrate.mp. or GTN.tw. [mp=title, abstract, original title, name of substance word, subject heading word, keyword heading word, protocol supplementary concept word, rare disease supplementary concept word, unique identifier, synonyms]

20. nitric Oxide Donors.tw

21. exp angiotensin-converting enzyme inhibitor/

22. exp ace inhibitor/

23. angiotensin-converting enzyme inhibitor$.mp

24. ace inhibitor$.mp

25. (alacepril or altiopril or benazepril or captopril or ceronapril or cilazapril or delapril or derapril or enalapril or fosinopril or idapril or imidapril or lisinopril or moexipril or moveltipril or pentopril or perindopril or quinapril or ramipril or spirapril or temocapril or randolapril or zofenopril).mp

26. exp Angiotensin Receptor Antagonists/

27. (angiotensin adj3 (receptor antagon$ or receptor block$)).tw

28. arb?.tw

29. (abitesartan or azilsartan or candesartan or elisartan or embusartan or eprosartan or forasartan or irbesartan or losartan or milfasartan or olmesartan or saprisartan or tasosartan or telmisartan or valsartan or zolasartan).mp

30. exp adrenergic beta-antagonists/

31. (beta adj2 (adrenergic? or antagonist? or block$ or receptor?)).tw

32. (acebutolol or alprenolol or amosulalol or arotinolol or atenolol or befunolol or betaxolol or bevantolol or bisoprolol or bopindolol or bucindolol or bucumolol or bufetolol or bufuralol or bunitrolol or bupranolol or butofilolol or carazolol or carteolol or carvedilol or celiprolol or cetamolol or cloranolol or cyanopindolol or deacetylmetipranolol or dihydroalprenolol or dilevalol or epanolol or esmolol or indenolol or iodocyanopindolol or labetalol or landiolol or levobunolol or mepindolol or metoprolol or metipranolol or moprolol or nadolol or nadoxolol

For peer review only - http://bmjopen.bmj.com/site/about/guidelines.xhtml

or nebivolol or nifenalol or nipradilol or oxprenolol or penbutolol or pindolol or practolol or pronethalol or propranolol or proxodolol or sotalol or sulfinalol or talinolol or tertatolol or tilisolol or timolol or toliprolol or xibenolol).mp

33. exp calcium channel blockers/

34. (calcium adj2 (inhibit$ or agonist? or exogenous or blockader?)).tw

35. (calcium channel blockers or amlodipine or amrinone or bencyclane or bepridil or cinnarizine or conotoxins or diltiazem or felodipine or fendiline or flunarizine or gallopamil or isradipine or lidoflazine or magnesium sulfate or mibefradil or nicardipine or nifedipine or nimodipine or nisoldipine or nitrendipine or perhexiline or prenylamine or verapamil or omega-agatoxin iva or omega-conotoxin gvia or omega-conotoxins).mp

36. exp diuretics/

37. (thiazide or furosemide or bumetanide or piretanide or torasemide or azosemide or ethacrynic acid or ticrynafen or trip amide or phenoxybenzoic acid or muzolimine or indacrinone or etozolin or ozolinone or cicletanine or cicletanine or tienilic acid or tizolemide or hydrochlorothiazide or chlorothiazide or buthiazide or bendroflumethiazide or hydroflumethiazide or trichlormethiazide or methylclothiazide or polythiazide or cyclothiazide or cyclopenthiazide or chlorthalidone or metolazone or quinethazone or fenquizone or clorexolone or clop amide or indapamide or diapamide or isodapamide or mefruside or xipamide or diuretic$).mp

38. exp adrenergic alpha antagonists/

39. (alpha adrenergic antagonist? or alpha adrenergic receptor antagonist? or adrenergic alpha antagonist?).tw

40. ((alpha or alpha-adrenergic) adj2 block$).tw

41. (alfuzosin or bunazosin or doxazosin or indoramin or metazosin or neldazosin or prazosin or silodosin or tamsulosin or terazosin or tiodazosin or trimazosin or urapidil or phentolamine or phenoxybenzamine).mp

42. (methyldopa or alphamethyldopa or amodopa or dopamet or dopegyt or dopegit or dopegite or emdopa or hyperpax or hyperpaxa or methylpropionic acid or dopergit or meldopa or methyldopate or medopa or medomet or sembrina or aldomet or aldometil or aldomin or hydopa or methyldihydroxyphenylalanine or methyl dopa or mulfasin or presinol

For peer review only - http://bmjopen.bmj.com/site/about/guidelines.xhtml

For peer review only

or presolisin or sedometil or sembrina or taquinil or dihydroxyphenylalanine or methylphenylalanine or methylalanine or alpha methyl dopa).mp.

43. (alazine or alphapress or apdormin or apresolin or apresolina or apresoline or apressin or apressoline or aprezin or clorana or depressan or deselazin or dihydralazine or dihydrallazine or dihydrazinophthalazin or dralzine or hidral or hydralacin or hydralazine or hydralazine or hydralizine or hydrallazin or hydralazine or hydrapres or hydrazinophtalizine or hydrazinophthalazine or hydrolazine or hypatol or hyperphen or hypoftalin or hypophthalin or idralazina or iopress or nepresol or nonpolin or novo-hylazin or plethorit or praeparat or resporidin or solesorin or tetrasoline or travinon).mp

44. exp nitroprusside/

45. aliskiren.mp

46. renin inhibitor$.mp

47. moxonidine.mp

48. (minoxidil or minoxidine).mp. [mp=title, abstract, original title, name of substance word, subject heading word, keyword heading word, protocol supplementary concept word, rare disease supplementary concept word, unique identifier, synonyms]

49. clonidine.mp

50. guanfacine.mp

51. 17 or 18 or 19 or 20 or 21 or 22 or 23 or 24 or 25 or 26 or 27 or 28 or 29 or 30 or 31 or 32 or 33 or 34 or 35 or 36 or 37 or 38 or 39 or 40 or 41 or 42 or 43 or 44 or 45 or 46 or 47 or 48 or 49 or 50

52. 16 or 51

53. randomized controlled trial/

54. randomization/

55. Clinical Trial/

56. (clin$ adj25 trial$).ti,ab

57. ((singl$ or doubl$ or trebl$ or tripl$) adj25 (blind$ or mask$)).ti,ab

58. placebo$.ti,ab

59. random$.ti,ab

For peer review only - http://bmjopen.bmj.com/site/about/guidelines.xhtml

For peer review only

60. 53 or 54 or 55 or 56 or 57 or 58 or 59

61. exp animals/ not humans.sh.

62. 60 not 61

63. 5 and 52 and 62

For peer review only

For peer review only - http://bmjopen.bmj.com/site/about/guidelines.xhtml

Embase search strategy

1. exp basal ganglia hemorrhage/ or intracranial hemorrhages/ or cerebral hemorrhage/ or intracranial hemorrhage, hypertensive/ or cerebrovascular disorders/

2. ((brain$ or cerebr$ or cerebell$ or intracerebral or intracran$ or parenchymal or intraparenchymal or intraventricular or infratentorial or supratentorial or basal gangli$ or putaminal or putamen or posterior fossa or hemispher$ or pon$ or lentiform$ or brainstem or cortic$ or cortex$ or subcortic$ or subcortex$) adj3 (h? emorrhag$ or h?ematoma$ or bleed$)).tw

3. ((hemorrhag$ or haemorrhag$) adj3 (stroke$ or apoplex$ or cerebral vasc$ or cerebrovasc$ or cva)).tw

4. (ICH or ICHs or PICH or PICHs).tw

5. 1 or 2 or 3 or 4

6. exp blood pressure/

7. exp hypertension/

8. (blood pressure or bloodpressure).tw

9. (hypertension or hypertensive).tw

10. ((bp or blood pressure) adj3 (lowering or reduc$)).tw

11. ((strict$ or target$ or tight$ or intens$ or below) adj3 (blood pressure or systolic or diastolic or bp or level$)).tw

12. ((manage$ or monitor$) adj3 (hypertension or blood pressure)).tw

13. ((intense or intensive or aggressive or accelerated or profound or radical or severe) adj3 ((bp or blood pressure) adj3 (lowering or reduc$ or decreas$ or decrement or dimin$ or declin$))).tw

14. ((standard or normal or ordinary or guideline or guide line or guideline recommend$ or recommend$ or convention$ or usual or established) adj3 ((bp or blood pressure) adj3 (lowering or reduc$ or decreas$ or decrement or dimin$ or declin$))).tw

15. (antihypertensive adj2 (agent$ or drug$ or medicat$)).tw

16. exp antihypertensive agents/

17. exp antihypertensive therapy/

For peer review only - http://bmjopen.bmj.com/site/about/guidelines.xhtml

18. 6 or 7 or 8 or 9 or 10 or 11 or 12 or 13 or 14 or 15 or 16 or 17

19. exp glyceryl trinitrate/

20. nitrate.tw

21. glyceryl trinitrate.mp. or GTN.tw

22. nitric Oxide Donors.tw

23. exp peptidyl carboxypeptidase inhibitor/

24. exp ace inhibitor/

25. angiotensin-converting enzyme inhibitor$.mp

26. ace inhibitor$.mp

27. (alacepril or altiopril or benazepril or captopril or ceronapril or cilazapril or delapril or derapril or enalapril or fosinopril or idapril or imidapril or lisinopril or moexipril or moveltipril or pentopril or perindopril or quinapril or ramipril or spirapril or temocapril or trandolapril or zofenopril).mp

28. exp Angiotensin Receptor Antagonists/

29. (angiotensin adj3 (receptor antagon$ or receptor block$)).tw

30. arb?.tw

31. (abitesartan or azilsartan or candesartan or elisartan or embusartan or eprosartan or forasartan or irbesartan or losartan or milfasartan or olmesartan or saprisartan or tasosartan or telmisartan or valsartan or zolasartan).mp

32. exp beta adrenergic receptor blocking agent/

33. (beta adj2 (adrenergic? or antagonist? or block$ or receptor?)).tw

34. (acebutolol or alprenolol or amosulalol or arotinolol or atenolol or befunolol or betaxolol or bevantolol or bisoprolol or bopindolol or bucindolol or bucumolol or bufetolol or bufuralol or bunitrolol or bupranolol or butofilolol or carazolol or carteolol or carvedilol or celiprolol or cetamolol or cloranolol or cyanopindolol or deacetylmetipranolol or dihydroalprenolol or dilevalol or epanolol or esmolol or indenolol or iodocyanopindolol or labetalol or landiolol or levobunolol or mepindolol or metoprolol or metipranolol or moprolol or nadolol or nadoxolol or nebivolol or nifenalol or nipradilol or oxprenolol or penbutolol or pindolol or practolol or

For peer review only - http://bmjopen.bmj.com/site/about/guidelines.xhtml

For peer review only

pronethalol or propranolol or proxodolol or sotalol or sulfinalol or talinolol or tertatolol or tilisolol or timolol or toliprolol or xibenolol).mp

35. exp calcium channel blocking agent/

36 (calcium adj2 (inhibit$ or agonist? or exogenous or blockader?)).tw

37. (calcium channel blockers or amlodipine or amrinone or bencyclane or bepridil or cinnarizine or conotoxins or diltiazem or felodipine or fendiline or flunarizine or gallopamil or isradipine or lidoflazine or magnesium sulfate or mibefradil or nicardipine or nifedipine or nimodipine or nisoldipine or nitrendipine or perhexiline or prenylamine or verapamil or omega-agatoxin iva or omegaconotoxin gvia or omega-conotoxins).mp

38. exp diuretic agent/

39. (thiazide or furosemide or bumetanide or piretanide or torasemide or azosemide or ethacrynic acid or ticrynafen or trip amide or phenoxybenzoic acid or muzolimine or indacrinone or etozolin or ozolinone or cicletanine or cicletanine or tienilic acid or tizolemide or hydrochlorothiazide or chlorothiazide or buthiazide or bendroflumethiazide or hydroflumethiazide or trichlormethiazide or methylclothiazide or polythiazide or cyclothiazide or cyclopenthiazide or chlorthalidone or metolazone or quinethazone or fenquizone or clorexolone or clop amide or indapamide or diapamide or isodapamide or mefruside or xipamide or diuretic$).mp

40. exp alpha adrenergic receptor blocking agent/

41. (alpha adrenergic antagonist? or alpha adrenergic receptor antagonist? or adrenergic alpha antagonist?).tw

42. ((alpha or alpha-adrenergic) adj2 block$).tw

43. (alfuzosin or bunazosin or doxazosin or indoramin or metazosin or neldazosin or prazosin or silodosin or tamsulosin or terazosin or tiodazosin or trimazosin or urapidil or phentolamine or phenoxybenzamine).mp

44. (methyldopa or alphamethyldopa or amodopa or dopamet or dopegyt or dopegit or dopegite or emdopa or hyperpax or hyperpaxa or methylpropionic acid or dopergit or meldopa or methyldopate or medopa or medomet or sembrina or aldomet or aldometil or aldomin or hydopa or methyldihydroxyphenylalanine or methyl dopa or mulfasin or presinol or presolisin or sedometil or sembrina or taquinil or dihydroxyphenylalanine or methylphenylalanine or methylalanine or alpha methyl dopa).mp

For peer review only

45. (alazine or alphapress or apdormin or apresolin or apresolina or apresoline or apressin or apressoline or aprezin or clorana or depressan or deselazin or dihydralazine or dihydrallazine or dihydrazinophthalazin or dralzine or hidral or hydralacin or hydralazine or hydralazine or hydralizine or hydrallazin or hydralazine or hydrapres or hydrazinophtalizine or hydrazinophthalazine or hydrolazine or hypatol or hyperphen or hypoftalin or hypophthalin or idralazina or iopress or nepresol or nonpolin or novo-hylazin or plethorit or praeparat or resporidin or solesorin or tetrasoline or travinon).mp

46. exp nitroprusside sodium/

47. aliskiren.mp

48. renin inhibitor$.mp

49. moxonidine.mp

50. (minoxidil or minoxidine).mp. [mp=title, abstract, original title, name of substance word, subject heading word, keyword heading word, protocol supplementary concept word, rare disease supplementary concept word, unique identifier] {Including Related Terms}

51. clonidine.mp

52. guanfacine.mp

53. 19 or 20 or 21 or 22 or 23 or 24 or 25 or 26 or 27 or 28 or 29 or 30 or 31 or 32 or 33 or 34 or 35 or 36 or 37 or 38 or 39 or 40 or 41 or 42 or 43 or 44 or 45 or 46 or 47 or 48 or 49 or 50 or 51 or 52

54. 18 or 53

55. crossover-procedure.sh

56. double-blind procedure.sh

57. single-blind procedure.sh

58. (random$ or factorial$ or crossover$ or cross over$ or cross-over$ or placebo$ or (doubl$ adj blind$) or (singl$ adj blind$) or assign$ or allocat$).tw,ot

59. randomized controlled trial.sh

60. trial.ti

61. controlled clinical trial/

62. 55 or 56 or 57 or 58 or 59 or 60 or 61

For peer review only - http://bmjopen.bmj.com/site/about/guidelines.xhtml

63. exp animal/ or exp invertebrate/ or animal.hw. or non human/ or nonhuman/

64. human/ or human cell/ or human tissue/ or normal human/

65. 63 not 64

66. 62 not 65

For peer review only

For peer review only - http://bmjopen.bmj.com/site/about/guidelines.xhtml

Cochrane Central Register of Controlled Trials

#1 MeSH descriptor: [Basal Ganglia Hemorrhage] explode all trees

#2 MeSH descriptor: [Intracranial Hemorrhage, Hypertensive] explode all trees

#3 MeSH descriptor: [Cerebral Hemorrhage] explode all trees

#4 MeSH descriptor: [Cerebrovascular Disorders] this term only

#5 (hemorrhag* or haemorrhag*) ti.ab.kw

#6 (stroke* or apoplex* or cerebral vasc*? or cerebrovasc* or cva) ti.ab.kw

#7 #5 and #6

#8 (brain* or cerebr* or cerebell* or intracerebral or intracran* or parenchymal or infratentorial or supratentorial or basal gangli*? or putaminal or putamen or posterior fossa? or pons or pontine or brainstem or lentiform) ti.ab.kw

#9 (haemorrhage* or hemorrhage* or haematoma* or hematoma* or bleed*) ti.ab.kw

#10 #8 and #9

#11 #1 or #2 or #3 or #4 or #7 or #10

#12 ((bp or blood pressure) adj5 (lowering or reduc$)) .tw

#13 ((strict$ or target$ or tight$ or intens$ or below) adj3 (blood pressure or systolic or diastolic or bp or level$)) .tw

#14 (hypertension or hypertensive) .tw

#15 ((manage$ or monitor$) adj3 (hypertension or blood pressure)) .tw

#16 ((intense or intensive or aggressive or accelerated or profound or radical or severe) adj5 ((bp or blood pressure) adj5 (lowering or reduc$ or decreas$ or decrement or dimin$ or declin $))) .tw

#17 ((standard or normal or ordinary or guideline or guide line or guideline recommend$ or recommend$ or convention$ or usual or established) adj5 ((bp or blood pressure) adj5 (lowering or reduc$ or decreas$ or decrement or dimin$ or declin$))) .tw

#18 (antihypertensive adj2 (agent$ or drug$ or medicat$)) .tw

#19 MeSH descriptor: [Antihypertensive Agents] explode all trees

#20 #12 or #13 or #14 or #15 or #16 or #17 or #18 or #19

For peer review only - http://bmjopen.bmj.com/site/about/guidelines.xhtml

#21 MeSH descriptor: [Angiotensin-Converting Enzyme Inhibitors] explode all trees

#22 angiotensin-converting enzyme inhibitor$.mp

#23 ace inhibitor$.mp

#24 (alacepril or altiopril or benazepril or captopril or ceronapril or cilazapril or delapril or derapril or enalapril or fosinopril or idapril or imidapril or lisinopril or moexipril or moveltipril or pentopril or perindopril or quinapril or ramipril or spirapril or temocapril or trandolapril or zofenopril) .mp

#25 MeSH descriptor: [Angiotensin Receptor Antagonists] explode all trees 2059

#26 (angiotensin adj3 (receptor antagon$ or receptor block $)) .tw

#27 arb?.tw

#28 (abitesartan or azilsartan or candesartan or elisartan or embusartan or eprosartan or forasartan or irbesartan or losartan or milfasartan or olmesartan or saprisartan or tasosartan or telmisartan or valsartan or zolasartan) .mp

#29 MeSH descriptor: [Adrenergic beta-Antagonists] explode all trees 4548

#30 (beta adj2 (adrenergic? or antagonist? or block$ or receptor?)) .tw

#31 (acebutolol or alprenolol or amosulalol or arotinolol or atenolol or befunolol or betaxolol or bevantolol or bisoprolol or bopindolol or bucindolol or bucumolol or bufetolol or bufuralol or bunitrolol or bupranolol or butofilolol or carazolol or carteolol or carvedilol or celiprolol or cetamolol or cloranolol or cyanopindolol or deacetylmetipranolol or dihydroalprenolol or dilevalol or epanolol or esmolol or indenolol or iodocyanopindolol or labetalol or landiolol or levobunolol or mepindolol or metoprolol or metipranolol or moprolol or nadolol or nadoxolol or nebivolol or nifenalol or nipradilol or oxprenolol or penbutolol or pindolol or practolol or pronethalol or propranolol or proxodolol or sotalol or sulfinalol or talinolol or tertatolol or tilisolol or timolol or toliprolol or xibenolol) .mp

#32 MeSH descriptor: [Calcium Channel Blockers] explode all trees

#33 (calcium adj2 (inhibit$ or agonist? or exogenous or blockader?)) .tw

#34 (calcium channel blockers or amlodipine or amrinone or bencyclane or bepridil or cinnarizine or conotoxins or diltiazem or felodipine or fendiline or flunarizine or gallopamil or isradipine or lidoflazine or magnesium sulfate or mibefradil or nicardipine or nifedipine or

For peer review only - http://bmjopen.bmj.com/site/about/guidelines.xhtml

nimodipine or nisoldipine or nitrendipine or perhexiline or prenylamine or verapamil or omega-agatoxin iva or omegaconotoxin gvia or omega-conotoxins) .mp

#35 MeSH descriptor: [Diuretics] explode all trees

#36 (thiazide or furosemide or bumetanide or piretanide or torasemide or azosemide or ethacrynic acid or ticrynafen or trip amide or phenoxybenzoic acid or muzolimine or indacrinone or etozolin or ozolinone or cicletanine or cicletanine or tienilic acid or tizolemide or hydrochlorothiazide or chlorothiazide or buthiazide or bendroflumethiazide or hydroflumethiazide or trichlormethiazide or methylclothiazide or polythiazide or cyclothiazide or cyclopenthiazide or chlorthalidone or metolazone or quinethazone or fenquizone or clorexolone or clop amide or indapamide or diapamide or isodapamide or mefruside or xipamide or diuretic$) .mp

#37 MeSH descriptor: [Adrenergic alpha-Antagonists] explode all trees

#38 (alpha adrenergic antagonist? or alpha adrenergic receptor

antagonist? or adrenergic alpha antagonist?) .tw

#39 ((alpha or alpha-adrenergic) adj2 block$) .tw

#40 (alfuzosin or bunazosin or doxazosin or indoramin or metazosin or neldazosin or prazosin or silodosin or tamsulosin or terazosin or tiodazosin or trimazosin or urapidil or phentolamine or phenoxybenzamine) .mp

#41 (methyldopa or alphamethyldopa or amodopa or dopamet or dopegyt or dopegit or dopegite or emdopa or hyperpax or hyperpaxa or methylpropionic acid or dopergit or meldopa or methyldopate or medopa or medomet or sembrina or aldomet or aldometil or aldomin or hydopa or methyldihydroxyphenylalanine or methyl dopa or mulfasin or presinol or presolisin or sedometil or sembrina or taquinil or dihydroxyphenylalanine or methylphenylalanine or methylalanine or alpha methyl dopa) .mp

#42 (alazine or alphapress or apdormin or apresolin or apresolina or apresoline or apressin or apressoline or aprezin or clorana or depressan or deselazin or dihydralazine or dihydrallazine or dihydrazinophthalazin or dralzine or hidral or hydralacin or hydralazine or hydralazine or hydralizine or hydrallazin or hydralazine or hydrapres or hydrazinophtalizine or hydrazinophthalazine or hydrolazine or hypatol or hyperphen or hypoftalin or hypophthalin or idralazina or iopress or nepresol or nonpolin or novo-hylazin or plethorit or praeparat or resporidin or solesorin or tetrasoline or travinon) .mp

For peer review only - http://bmjopen.bmj.com/site/about/guidelines.xhtml

#43 MeSH descriptor: [Nitroprusside] explode all trees

#44 aliskiren.mp

#45 renin inhibitor$.mp

#46 moxonidine.mp

#47 (minoxidil or minoxidine) .mp

#48 clonidine.mp

#49 guanfacine.mp

#50 MeSH descriptor: [Nitroglycerin] explode all trees

#51 #21 or #22 or #23 or #24 or #25 or #26 or #27 or #28 or #29 or #30 or #31 or #32 or #33 or #34 or #35 or #36 or #37 or #38 or #39 or #40 or #41 or #42 or #43 or #44 or #45 or #46 or #47 or #48 or #49 or #50 17095

#52 #20 or #51 23976

#53 #11 and #52 306

For peer review only - http://bmjopen.bmj.com/site/about/guidelines.xhtml

SUPPLEMENTARY MATERIAL 2: Variables for extraction

Trial information	**Name of trial/acronym, chief investigator/study group, years conducted, number of participants randomised, number of participants treated (intervention vs. controls), number of participants with primary outcome.**
Demographics	**Age, sex,** ethnicity**, country**.
Medical history [Y/N]	Premorbid disability (mRS), **hypertension**, diabetes mellitus, ischaemic stroke, intracerebral haemorrhage, transient ischaemic attack, ischaemic heart disease including angina and myocardial infarction, congestive cardiac failure, atrial fibrillation, valvular heart disease, peripheral arterial disease, hyperlipidaemia, family history of young stroke, current alcohol excess, smoker (active/previous/never).
Medications at time of admission [Y/N]	**BP lowering therapy**: ACEi, ARB, BB, CCB, diuretic, AB, nitric oxide donor, centrally acting, other<br>**Anticoagulant therapy**: warfarin or non-VKA oral anticoagulant<br>**Antiplatelet therapy**: aspirin, clopidogrel, cilostazol, dipyridamole, other, two or more<br>Lipid lowering therapy: statin, other
Baseline clinical variables	**Systolic BP [mmHg], diastolic BP [mmHg], heart rate [beats per min],** presence of AF on electrocardiogram [Y/N], dysphagia [Y/N], **NIHSS score [/42], GCS score [/15]**, Scandinavian Neurological Stroke Scale [/58], stroke syndrome [TACS/PACS/LACS/POCS].
Baseline blood tests	eGFR [mL/min], creatinine [µmol/L or mg/dL], urea [mmol/L], sodium [mmol/L], potassium [mmol/L] blood glucose [mmol/L or mg/dL], osmolarity [calculated].
Baseline neuroimaging characteristics	**Scan availability [Y/N]**, CT [Y/N], CT-A [Y/N], MRI [Y/N], **ICH volume [mm^3 or mL], ICH location** [lobar or basal ganglia/deep or infratentorial/brainstem/cerebellar], **presence of intraventricular haemorrhage [Y/N]**, IVH volume [mm^3 or mL], CT-A spot sign [Y/N], mass effect [Y/N], perihaematomal oedema volume [mm^3 or mL], small vessel disease [Y/N], brain atrophy [Y/N], evidence of old stroke [Y/N].
BP treatment	**Time from onset to treatment [hours:minutes], treatment group [intensive vs. standard or intervention vs. control],** route of administration [IV/oral/transdermal/sublingual]

For peer review only - http://bmjopen.bmj.com/site/about/guidelines.xhtml

	IV antihypertensive agents: BB, CCB, diuretic, AB, nitric oxide donor, nitroprusside, hydralazine, other –name [Y/N]<br>Transdermal agents: GTN patch [Y/N]<br>Oral antihypertensive agent: ACEi, ARB, BB, CCB, diuretic, AB, central acting, other – name [Y/N]<br>Time to achieve target BP [hours:minutes]<br>**All trial BP and heart rate measurements within first 168 hours.**
Other management [Y/N]	Intubation, ICU/ITU, surgery [evacuation/ intraventricular drainage/ minimally invasive surgery/ other – name], VTE prophylaxis [heparin/ compression stockings/ intermittent pneumatic compression], mannitol, haemostatic agents, DNAR/withdrawal of active treatment.
Outcomes	**Death: within 90 days [Y/N]**<br>**Disability: mRS at day 7 and 90 [0-6],** NIHSS score at days 2, 3 and 7, Barthel index at day 7, 90, 180.<br>Quality of life: 5-level EQ5D score at 90 days [/1]<br>Cognitive function: MMSE at 90 days [/30]<br>**Serious adverse events: all fatal and non-fatal,** renal, VTE, pneumonia**, early neurologic deterioration, symptomatic hypotension requiring corrective therapy**<br>Haematoma characteristics on repeat brain imaging: ICH volume [mm^3 or mL], IVH volume [mm^3 or mL], perihaematomal oedema volume [mm^3 or mL]

Bold text indicates core variable

mRS indicates modified Rankin Scale; ACEi, angiotensin-converting enzyme inhibitor; ARB, angiotensin receptor blocker; BB, beta blocker; CCB, calcium channel blocker; AB, alpha blocker; BP, blood pressure; AF, atrial fibrillation; NIHSS, National Institute for Health Stroke Scale; GCS, Glasgow coma scale; TACS, total anterior circulation syndrome; PACS, partial anterior circulation syndrome; LACS, lacunar syndrome; POCS, posterior circulation syndrome; eGFR, estimated glomerular filtration rate; CT, computed tomography; CT-A, computed tomographic angiography; MRI, magnetic resonance imaging; ICH, intracerebral haemorrhage; IV, intravenous; GTN, glyceryl trinitrate; ICU/ITU, intensive care/treatment unit; VTE, venous thromboembolism; DNAR, do not attempt resuscitation, EQ-5D EuroQOL five dimensions health-related quality of life questionnaire

For peer review only

SUPPLEMENTARY MATERIAL 3: Table shells

TABLE 1. Baseline characteristics according to treatment group. Data are number (%), mean (standard deviation) or median (interquartile range), chi-squared or Kruskal-Wallis tests will be used to make comparisons.

	Active	Control	P
Age (years)			
Sex, female			
Geographical region			
North America			
Asia			
Europe			
Other			
ICH onset to randomisation (hrs)			
Medical history			
Hypertension			
Diabetes mellitus			
Stroke			
Ischaemic heart disease			
AF (history or ECG finding)			
Smoker			
Medication history			
Antihypertensive			
Antiplatelet			
Anticoagulation			
Clinical			
Systolic BP (mmHg)			
Diastolic BP (mmHg)			
Pulse pressure (mmHg)			
Heart rate (bpm)			

For peer review only

Rate-pressure product[1]
NIHSS (/42)
GCS (/15)
Neuroimaging
Haematoma volume (ml)
Haematoma location
Lobar
Basal ganglia/deep
Infratentorial
Intraventricular haemorrhage
Perihaematomal oedema
Absolute (ml)
Relative[2]
Pre-stroke characteristics
Leukoaraiosis
Atrophy
Prior stroke lesion

ICH denotes intracerebral haemorrhage; AF, atrial fibrillation; BP, blood pressure; NIHSS, National Institute for Health stroke scale; GCS, Glasgow come scale

[1]Systolic BP multiplied by heart rate

[2]Perihaematomal oedema to intraparenchymal haemorrhage volume ratio

For peer review only

TABLE 2. Treatment used to lower blood pressure, and other processes of care. Data are number (%). Chi squared tests will be used to make comparisons.

	Treatment	Control	P
Route of administration			
Oral			
Intravenous			
Transdermal			
Sublingual			
BP intervention ^			
Target *			
ACE-I, po/sl §			
ARA, po +			
ß-RA, po/im §§			
CCB, iv ¥			
CCB, po $			
Nitrate, topical #			
Magnesium, iv ∫			
Other treatments (%)			
Mannitol			
Haemostatic agents			
VTE prophylaxis			
DNAR order/withdrawal of active treatment			
ITU/intubation			
Neurosurgery			

BP denotes blood pressure; ACE-I, angiotensin converting enzyme inhibitor; ARA, angiotensin receptor antagonist; ß-RA, beta receptor antagonist; CCB, calcium channel blocker; VTE, venous thromboembolism; DNAR, do not attempt resuscitation; ITU, intensive treatment unit; po, oral; sl, sublingual; im, intramuscular; iv, intravenous

^ Categorised according to current BASC collaborator trials

*INTERACT1&2, ATACH-II, ICH-ADAPT; § CHHIPS, ACEi arm; + SCAST; §§ CHHIPS, ß-RA arm; ¥ ATACH-II, Uzuner; $ VENUS; # GTN1&2, ENOS, RIGHT; ∫ IMAGES, FAST-Mag

For peer review only

For peer review only - http://bmjopen.bmj.com/site/about/guidelines.xhtml

TABLE 3. Effect of BP lowering on outcomes. Data are number (%), mean (standard deviation) or median (interquartile range); mean difference (MD) or odds ratios (OR) with 95% confidence intervals (CI). Generalised linear mixed models for binary, continuous and ordinal outcomes, with random intercept for source trial. Multivariable analyses adjusted for age, sex, severity (NIHSS), systolic BP, ICH volume, presence of IVH and time to randomisation.

	N	Treatment	Control	OR/MD	95% CI	P	OR/MD	95% CI	P
				Unadjusted			Adjusted		
**On treatment outcomes**									
Within 1 hour of randomisation									
Systolic BP (mmHg)									
Mean[1]				MD			MD		
Delta[2]				MD			MD		
Peak[3]				MD			MD		
Standard deviation[4]				MD			MD		
Diastolic BP § (mmHg)				MD			MD		
Pulse pressure § (mmHg)				MD			MD		
Heart rate § (bpm)				MD			MD		
Rate pressure product[5]				MD			MD		
Within 24 hours of randomisation									
Systolic BP (mmHg)									

For peer review only - http://bmjopen.bmj.com/site/about/guidelines.xhtml

Mean[1]	MD	MD
Delta[2]	MD	MD
Peak[3]	MD	MD
Standard deviation[4]	MD	MD
Diastolic BP § (mmHg)	MD	MD
Pulse pressure § (mmHg)	MD	MD
Heart rate § (bpm)	MD	MD
Rate pressure product[5]	MD	MD
Neurologic deterioration	OR	OR
Symptomatic hypotension	OR	OR
Within 7 days of randomisation		
Systolic BP (mmHg)		
Mean[1]	MD	MD
Delta[2]	MD	MD
Peak[3]	MD	MD
Standard deviation[4]	MD	MD
Diastolic BP § (mmHg)	MD	MD
Pulse pressure § (mmHg)	MD	MD
Heart rate § (bpm)	MD	MD

For peer review only - http://bmjopen.bmj.com/site/about/guidelines.xhtml

Rate pressure product[5]	MD	MD
Neurologic deterioration	OR	OR
Symptomatic hypotension	OR	OR
**Repeat brain imaging**		
Completed within 48 hours	OR	OR
Haematoma volume		
Absolute (ml)	MD	MD
Expansion		
>33%	OR	OR
>6ml	OR	OR
Perihaematomal oedema volume		
Absolute (ml)	MD	MD
Relative[6]	MD	MD
Mass effect	OR	OR
**90-day clinical outcomes**		
Primary outcome (unfavourable shift in ordinal mRS score)	OR	OR
Death (%)	OR	OR

For peer review only - http://bmjopen.bmj.com/site/about/guidelines.xhtml

Dichotomised mRS score		
4-6, death and major disability (%)	OR	OR
3-6, death and dependency (%)	OR	OR
Barthel index (/100)	MD	MD
EQ-5D-5L (/25)	MD	MD
MMSE (/30)	MD	MD
Serious adverse events		
Fatal	OR	OR
Non-fatal	OR	OR
Renal	OR	OR
Cardiac	OR	OR
Pneumonia	OR	OR
VTE	OR	OR

BP denotes blood pressure; SD, standard deviation; bpm, beats per minute; mRS, modified Rankin Scale; EQ-5D-5L, European Quality of Life–5 Dimensions 5-level questionnaire; MMSE, mini mental state examination; VTE, venous thromboembolism.

[1]Mean of measures within specified period

[2]Baseline minus mean within specified period

[3]Highest value within specified period

[4]Standard deviation of measures within specified period

[5]Peak systolic BP multiplied by peak heart rate within specified period

§ Mean, delta, peak and standard deviation will also be assessed

For peer review only - http://bmjopen.bmj.com/site/about/guidelines.xhtml

FIGURE 1. Shift in mRS according to BP lowering vs. control/guideline treatment. Give adjusted odds ratio from generalised linear mixed model with source trial as random effect. Adjusted for age, sex, severity (NIHSS), systolic BP, ICH volume at baseline, IVH at baseline, time to randomisation.

For peer review only

For peer review only - http://bmjopen.bmj.com/site/about/guidelines.xhtml

FIGURE 2. Forest plot of shift in mRS by pre-defined subgroups. Same model as figure 1 with interaction tests.

- Age: ≤70, >70 (years)
- Sex
- Region: Asian vs other
- Pre-stroke hypertension: Y/N
- Pre-stroke antithrombotic use: Y/N
- Baseline SBP: ≤140, 140-180, 180-220, >220 (mmHg)
- NIHSS: ≤ 8, 9-15, >15
- Haematoma volume at baseline: ≤ 15, >15 (ml)
- Type of intervention: target vs angiotensin inhibitor vs CCB vs NO donor vs magnesium/other
- Time from stroke to randomisation: <2, 2-6, 6-48, >48 (hours)
- Type of trial: pre-hospital vs hospital, ICH only vs mixed

For peer review only

For peer review only - http://bmjopen.bmj.com/site/about/guidelines.xhtml

FIGURE 3. Kaplan-Meier curve for death with comparison of active and control treatment groups. Model adjusted for age, sex, severity (NIHSS), systolic BP, ICH volume at baseline, IVH at baseline, time to randomisation and source trial.

For peer review only

For peer review only - http://bmjopen.bmj.com/site/about/guidelines.xhtml

FIGURE 4. Forest plot for end-of-trial death by pre-defined subgroups. Same model as figure 1 with interaction tests.

- Age: ≤70, >70 (years)
- Sex
- Region: Asian vs other
- Pre-stroke hypertension: Y/N
- Pre-stroke antithrombotic use: Y/N
- Baseline SBP: ≤140, 140-180, 180-220, >220 (mmHg)
- NIHSS: ≤ 8, 9-15, >15
- Haematoma volume at baseline: ≤ 15, >15 (ml)
- Type of intervention: target vs angiotensin inhibitor vs CCB vs NO donor vs magnesium/other
- Time from stroke to randomisation: <2, 2-6, 6-48, >48 (hours)
- Type of trial: pre-hospital vs hospital, ICH only vs mixed

For peer review only

For peer review only - http://bmjopen.bmj.com/site/about/guidelines.xhtml

FIGURE 5. Meta-regression plots: relationship between odds ratio for unfavourable shift in scores on the modified Rankin Scale at end of trial and (i) time to randomisation; (ii) baseline systolic blood pressure; (iii) baseline haematoma volume; (iv) mean achieved systolic blood pressure 24 hours post-randomisation; and (v) change in systolic blood pressure at 1 hour or soonest trial measure thereafter.[1,2] Model (i) is adjusted for age, sex, severity, type of treatment, and source trial; models (ii)-(iv) are adjusted for the same plus time to randomisation.

For peer review only

REFERENCES

1 Leonardi-Bee J, Bath PMW, Phillips SJ, Sandercock PAG. Blood pressure and clinical outcomes in the International Stroke Trial. *Stroke* 2002. DOI:10.1161/01.STR.0000014509.11540.66.

2 Saver JL, Goyal M, Van Der Lugt A, *et al.* Time to treatment with endovascular thrombectomy and outcomes from ischemic stroke: A meta-analysis. *JAMA - J Am Med Assoc* 2016. DOI:10.1001/jama.2016.13647.

For peer review only

For peer review only - http://bmjopen.bmj.com/site/about/guidelines.xhtml

Reporting checklist for protocol of a systematic review.

Based on the PRISMA-P guidelines.

Instructions to authors

Complete this checklist by entering the page numbers from your manuscript where readers will find each of the items listed below.

Your article may not currently address all the items on the checklist. Please modify your text to include the missing information. If you are certain that an item does not apply, please write "n/a" and provide a short explanation.

Upload your completed checklist as an extra file when you submit to a journal.

In your methods section, say that you used the PRISMA-P reporting guidelines, and cite them as:

Moher D, Shamseer L, Clarke M, Ghersi D, Liberati A, Petticrew M, Shekelle P, Stewart LA. Preferred Reporting Items for Systematic Review and Meta-Analysis Protocols (PRISMA-P) 2015 statement. Syst Rev. 2015;4(1):1.

		Reporting Item	Page Number
Identification	#1a	Identify the report as a protocol of a systematic review	1
Update	#1b	If the protocol is for an update of a previous systematic review, identify as such	n/a
	#2	If registered, provide the name of the registry (such as	5

For peer review only

		PROSPERO) and registration number	
Contact	#3a	Provide name, institutional affiliation, e-mail address of all protocol authors; provide physical mailing address of corresponding author	1
Contribution	#3b	Describe contributions of protocol authors and identify the guarantor of the review	14
	#4	If the protocol represents an amendment of a previously completed or published protocol, identify as such and list changes; otherwise, state plan for documenting important protocol amendments	
Sources	#5a	Indicate sources of financial or other support for the review	14
Sponsor	#5b	Provide name for the review funder and / or sponsor	14
Role of sponsor or funder	#5c	Describe roles of funder(s), sponsor(s), and / or institution(s), if any, in developing the protocol	14
Rationale	#6	Describe the rationale for the review in the context of what is already known	4
Objectives	#7	Provide an explicit statement of the question(s) the review will address with reference to participants, interventions, comparators, and outcomes (PICO)	5
Eligibility criteria	#8	Specify the study characteristics (such as PICO, study design, setting, time frame) and report characteristics (such as years considered, language, publication status) to be used as criteria for eligibility for the review	6

For peer review only

Information sources	#9	Describe all intended information sources (such as electronic databases, contact with study authors, trial registers or other grey literature sources) with planned dates of coverage	7
Search strategy	#10	Present draft of search strategy to be used for at least one electronic database, including planned limits, such that it could be repeated	7
Study records - data management	#11a	Describe the mechanism(s) that will be used to manage records and data throughout the review	7
Study records - selection process	#11b	State the process that will be used for selecting studies (such as two independent reviewers) through each phase of the review (that is, screening, eligibility and inclusion in meta-analysis)	7
Study records - data collection process	#11c	Describe planned method of extracting data from reports (such as piloting forms, done independently, in duplicate), any processes for obtaining and confirming data from investigators	8
Data items	#12	List and define all variables for which data will be sought (such as PICO items, funding sources), any pre-planned data assumptions and simplifications	8, sup. material 2
Outcomes and prioritization	#13	List and define all outcomes for which data will be sought, including prioritization of main and additional outcomes, with rationale	9
Risk of bias in	#14	Describe anticipated methods for assessing risk of bias of	10

For peer review only

individual studies		individual studies, including whether this will be done at the outcome or study level, or both; state how this information will be used in data synthesis	
Data synthesis	#15a	Describe criteria under which study data will be quantitatively synthesised	10
	#15b	If data are appropriate for quantitative synthesis, describe planned summary measures, methods of handling data and methods of combining data from studies, including any planned exploration of consistency (such as I2, Kendall's τ)	11, sup. material 3
	#15c	Describe any proposed additional analyses (such as sensitivity or subgroup analyses, meta-regression)	11, sup. material 3
	#15d	If quantitative synthesis is not appropriate, describe the type of summary planned	n/a
Meta-bias(es)	#16	Specify any planned assessment of meta-bias(es) (such as publication bias across studies, selective reporting within studies)	11
Confidence in cumulative evidence	#17	Describe how the strength of the body of evidence will be assessed (such as GRADE)	11

The PRISMA-P checklist is distributed under the terms of the Creative Commons Attribution License CC-BY 4.0. This checklist was completed on 08. May 2018 using http://www.goodreports.org/, a tool made by the EQUATOR Network in collaboration with Penelope.ai

For peer review only

BMJ Open

Lowering blood pressure after acute intracerebral haemorrhage: protocol for a systematic review and meta-analysis using individual patient data from randomised controlled trials participating in the Blood pressure in Acute Stroke Collaboration (BASC)

Journal:	*BMJ Open*
Manuscript ID	bmjopen-2019-030121.R1
Article Type:	Protocol
Date Submitted by the Author:	02-May-2019
Complete List of Authors:	Moullaali, Tom; University of Edinburgh, Centre for Clinical Brain Sciences; George Institute for Global Health<br>Wang, Xia; George Institute for Global Health<br>Woodhouse, Lisa; University of Nottingham, Stroke Trials Unit<br>Law, Zhe Kang; University of Nottingham, Stroke Trials Unit; National University of Malaysia<br>Delcourt, Candice; George Institute for Global Health; Royal Prince Alfred Hospital<br>Sprigg, Nikola; University of Nottingham, Stroke Trials Unit; Nottingham University Hospitals NHS Trust, Stroke<br>Krishnan, Kailash; University of Nottingham, Stroke Trials Unit; Nottingham University Hospitals NHS Trust, Stroke<br>Robinson, Thompson; University of Leicester, Department of Cardiovascular Sciences and NIHR Leicester Biomedical Research Centre<br>Wardlaw, Joanna; University of Edinburgh, Centre for Clinical Brain Sciences<br>Salman, Rustam; University of Edinburgh, Centre for Clinical Brain Sciences<br>Berge, Eivind; Oslo University Hospital, Department of Internal Medicine<br>Sandset, Else; Oslo University Hospital, Neurology Department; Norwegian Air Ambulance Foundation, Research and Development<br>Anderson, Craig; George Institute for Global Health; Royal Prince Alfred Hospital, Neurology Department<br>Bath, Philip; University of Nottingham, Stroke Trials Unit; Nottingham University Hospitals NHS Trust, Stroke
**Primary Subject Heading**:	Neurology
Secondary Subject Heading:	Evidence based practice, Medical management
Keywords:	Stroke < NEUROLOGY, intracerebral haemorrhage, blood pressure lowering, individual participant data meta-analysis

Lowering blood pressure after acute intracerebral haemorrhage: protocol for a systematic review and meta-analysis using individual patient data from randomised controlled trials participating in the Blood pressure in Acute Stroke Collaboration (BASC)

Authors

The BASC Investigators*

*Writing Committee

Tom J Moullaali†,[1,2] Xia Wang†,[1] Lisa J Woodhouse,[3] Zhe Kang Law,[3,4,5] Candice Delcourt,[1,6] Nikola Sprigg,[3,4] Kailash Krishnan,[3,4] Thompson G Robinson,[7] Joanna Wardlaw,[2] Rustam Al-Shahi Salman,[2] Eivind Berge,[8] Else Charlotte Sandset,[9,10] Craig S Anderson,[1,6,11] Philip M Bath.[3,4]

[1]The George Institute for Global Health, Faculty of Medicine, University of New South Wales, NSW, Australia

[2]Centre for Clinical Brain Sciences, University of Edinburgh, University of Edinburgh, Edinburgh, UK

[3]Stroke Trials Unit, Division of Clinical Neuroscience, University of Nottingham, City Hospital Campus, Nottingham, UK

[4]Stroke, Nottingham University Hospitals NHS Trust, Nottingham, UK

[5]National University of Malaysia, Kuala Lumpur, Malaysia

[6]Neurology Department, Royal Prince Alfred Hospital, The University of Sydney, Sydney, NSW, Australia

[7]University of Leicester, Department of Cardiovascular Sciences and NIHR Leicester Biomedical Research Centre, Leicester, UK

[8]Department of Internal Medicine, Oslo University Hospital, Oslo, Norway

[9]Department of Neurology, Oslo University Hospital, Postboks 4956 Nydalen 0424 Oslo, Norway

[10]Research and Development Department, The Norwegian Air Ambulance Foundation, Oslo, Norway

For peer review only

[11]The George Institute China at Peking University Health Science Center, Beijing, PR China

†Co-first authors

Corresponding Author:

Professor Craig S Anderson

The George Institute for Global Health

PO Box M201, Missenden Road, NSW 2050, AUSTRALIA

T: +61-2-9993-4500; F: +61-2-9993-4502

canderson@georgeinstitute.org.au

Email addresses for writing committee:

tom.moullaali@ed.ac.uk

xwang@georgeinstitute.org.au

l.woodhouse@nottingham.ac.uk

zhe.law@nottingham.ac.uk

cdelcourt@georgeinstitute.org.au

nikola.sprigg@nottingham.ac.uk

kailash.krishnan@nottingham.ac.uk

tgr2@leicester.ac.uk

joanna.wardlaw@ed.ac.uk

rustam.al-shahi@ed.ac.uk

eivind.berge@medisin.uio.no

else@sandset.net

philip.bath@nottingham.ac.uk

Word count abstract: 252

Word count body: 2224

Tables: 0

Supplementary materials: 3

Figures: 0

Key words: acute stroke; intracerebral haemorrhage; blood pressure lowering; hypertension; modified Rankin Scale

For peer review only - http://bmjopen.bmj.com/site/about/guidelines.xhtml

Abstract

Introduction

Conflicting results from multiple randomised trials indicate that the methods and effects of blood pressure (BP) reduction after acute intracerebral haemorrhage (ICH) are complex. The Blood pressure in Acute Stroke Collaboration (BASC) is an international collaboration which aims to determine the optimal management of BP after acute stroke including ICH.

Methods and analysis

A systematic review will be undertaken according to the Preferred Reporting Items for Systematic review and Meta-Analysis of Individual Participant Data (PRISMA-IPD) guideline. A search of Cochrane Central Register of Controlled Trials, EMBASE, and MEDLINE from inception will be conducted to identify randomised controlled trials of blood pressure management in adults with acute spontaneous (non-traumatic) ICH enrolled within the first 7 days of symptom onset will be included. Authors of studies that meet the inclusion criteria will be invited to share their IPD. The primary outcome will be functional outcome according to the modified Rankin Scale. Safety outcomes will be early neurologic deterioration, symptomatic hypotension and serious adverse events. Secondary outcomes will include death, and neuroradiological and haemodynamic variables. Meta-analyses of pooled IPD using the intention-to-treat dataset of included trials, including subgroup analyses to assess modification of the effects of BP lowering by time to treatment, treatment strategy, and patient demographic, clinical and pre-stroke neuroradiological characteristics.

Ethics and dissemination

No new patient data will be collected nor is there any deviation from the original purposes of each study where ethical approvals were granted, therefore further ethical approval is not required. Results will be reported in international peer-reviewed journals.

For peer review only

Article summary

Strengths and limitations of this study:

- The Blood pressure in Acute Stroke Collaboration (BASC) is an international collaboration with the prospective aim of pooling individual participant data (IPD) from randomised controlled trials (RCTs) of blood pressure (BP) management in acute stroke, including intracerebral haemorrhage (ICH).
- Meta-analysis of IPD is regarded as the gold standard for synthesising evidence from multiple RCTs.
- This study aims to collect sufficient data to determine the optimal management of BP in acute ICH and will facilitate comparisons across subgroups according to patient characteristics, time to treatment and treatment strategy.
- This study will identify associations of treatment-related haemodynamic parameters which will inform future research.

Introduction

Rationale

Acute treatments proven to alter the prognosis of stroke due to spontaneous (non-traumatic) intracerebral haemorrhage (ICH) are limited. A 2014 Cochrane review that appraised the totality of evidence for blood pressure (BP) lowering within one week of acute stroke concluded that the supporting evidence was insufficient to make clear recommendations about this intervention, and that the findings were similar for ischaemic stroke and ICH.[1] In regard to ICH, several randomised controlled trials (RCTs) have focussed on early intensive BP reduction and included participants with mild to moderate acute ICH in the hospital setting.[2–4] Other trials commenced BP lowering in the pre-hospital period before diagnostic brain imaging, and by their nature included a mixture of ischaemic stroke and ICH patients, including more severe cases of ICH.[5–7] Data from ICH cases are also available from RCTs which used longer inclusion windows (up to 48 hours).[8–12] Furthermore, various agents and BP targets have been tested, and few trials considered prognostic implications of pre-stroke neuroimaging characteristics. Therefore, a substantial and varied body of evidence about BP lowering and outcome after acute ICH is available, but much of it is conflicting.[13]

The Blood pressure in Acute Stroke Collaboration (BASC) is an international collaboration which aims to prospectively pool individual participant data (IPD) from RCTs of BP control

For peer review only - http://bmjopen.bmj.com/site/about/guidelines.xhtml

after acute stroke, including stroke due to ICH.[14,15] IPD meta-analysis is considered the gold-standard for synthesising evidence from RCTs[16] and in this context, provides added value to meta-analyses of aggregate data[17] by facilitating multivariable analyses of treatment effects and subgroup analyses according to time to treatment, treatment strategy used, and baseline characteristics including neurological severity and haematoma volume, adjusted for confounding factors.

Primary objective

Determine the effect of BP lowering on clinical outcomes in patients with acute ICH and elevated BP.

Secondary objectives

i) Determine the effect of BP lowering according to baseline patient clinical and neuroradiological characteristics, BP lowering strategy, and timing of the intervention.

ii) Determine the effect of BP lowering on radiological outcomes.

iii) Where sufficient data are available, report associations of on-treatment haemodynamic parameters, adjusted for known confounders.

Methods and analysis

This systematic review and IPD meta-analysis will be performed in accordance with the recommendations made by the methods group of the Cochrane Collaboration (http://ipdmamg.cochrane.org/resources) and the UK Medical Research Council Network of Hubs for Trials Methodology Research.[18] The protocol has been developed in accordance with the Preferred Reporting Items for Systematic reviews and Meta-Analyses of IPD (PRISMA-IPD) checklist,[19] and will be registered with the international prospective register of systematic reviews, PROSPERO.[20]

Patient and public involvement

This protocol was developed without the involvement of patients or members of the public.

Eligibility criteria

Study designs

RCTs of BP reduction during the acute phase (<7 days) of stroke will be included.

Participants

For peer review only - http://bmjopen.bmj.com/site/about/guidelines.xhtml

Adults (>18 years) with spontaneous ICH will be included. For trials including both ICH and ischaemic stroke, we will include only patients with ICH. Participants with secondary ICH (e.g. due to trauma, tumour or vascular malformation) will be excluded. Uncertainty about an individual trial's eligibility criteria will be clarified with the appropriate investigator.

Interventions

Trials which involve interventions that lower BP including oral, sublingual, transdermal and intravenous agents, and single or combination therapy, will be considered.

Comparators

Control groups will typically be managed with placebo or according to contemporaneous guideline recommendations.

Outcomes

Trials that report data on death and functional outcome using the seven-level modified Rankin Scale (mRS, where scores range from 0 = no symptoms to 5 = severe disability, and 6 = death) at 90 days will be included. Where outcome data are not reported this way, the nearest time-point or suitable surrogate will be considered. Data regarding other outcomes (survival, quality of life, cognitive function), serious adverse events (SAEs), haematoma characteristics (growth, final volume, mass effect, oedema), and haemodynamic measures will be sought.

Setting

There will be no restrictions on the type of setting.

Language

There will be no language restriction.

Information sources

Trials will be sought using electronic searches of Cochrane Central Register of Controlled Trials, EMBASE, and MEDLINE, and in the reference lists of published systematic reviews and *ad hoc* reviews. Ongoing or unpublished trials will be identified using ClinicalTrials.gov and the World Health Organisation's International Clinical Trials Registry Platform (ICTRP).

Search strategy

Searches of the above bibliographic databases will be conducted using a combination of search terms relevant to the proposed study (supplementary material 1). Databases will be searched from inception to the present. The search strategy will be enhanced through searches

For peer review only - http://bmjopen.bmj.com/site/about/guidelines.xhtml

For peer review only

of the existing BASC reviews, the authors' publication databases, and reference lists in identified articles.

Selection process

All titles and abstracts will be screened against the eligibility criteria by TJM and ZKL. Any disagreements will be settled by an adjudicator, PMB. The principal investigators of eligible studies will be invited to participate in the collaboration.

Data management

Data sharing

Investigators of eligible studies will receive a written invitation to share their IPD. To ensure transparency, collaborators sharing data with BASC will be asked to sign a data transfer agreement for the predefined and appropriate use of their data according to this protocol.

Data checking

Initial internal analyses will compare data from each trial with their published results to ensure that data are complete and transferred without error: this will include checking the primary outcome, and all baseline variables or secondary outcomes relevant to the proposed analyses. The integrity of all data will be checked and any queries resolved with individual trial investigators.

Data merging

Datasets obtained from collaborating studies will be combined to form a new master dataset, which will include a variable to indicate the original study.

Confidentiality, data storage and handling

Each collaborator will de-identify their dataset to ensure no patient identifiable information is transferred. Data will be shared electronically, stored on password-protected, encrypted hard disks in a locked room, with daily backup facilitating disaster recovery.[15] Data will not be shared with anyone outside the collaborating group.

Data items

Variables that will be requested from participating investigators are listed in supplementary material 2, and can be outlined as follows:

- Trial information
- Demographics

For peer review only

- Medical history
- Medications at time of admission
- Baseline clinical variables
- Baseline neuroimaging characteristics of the acute ICH and pre-stroke features (leukoaraiosis, atrophy, prior stroke lesions)
- BP treatment and all trial BP data
- Clinical and radiological outcome data

Study outcomes

Primary outcome

The preferred primary effect variable will be functional outcome defined by the ordinal distribution of mRS scores at the end of trial follow-up (usually 3 months).

Secondary outcomes

(i) Death and dependency (3-6 on the mRS); (ii) death or severe disability (4-6 on the mRS) (iii) all-cause death; (iv) health-related quality of life (mobility, self-care, usual activities, pain or discomfort, and anxiety or depression), as assessed with the use of the European Quality of Life–5 Dimensions (EQ-5D) questionnaire.

Safety outcomes

(i) Early neurologic deterioration (as defined by each individual trial); (ii) symptomatic hypotension (as defined by each individual trial); (iii) other SAEs, as defined by trial, to include fatal, non-fatal and treatment-related SAEs (including renal SAEs).

Radiological outcomes

(i) Haematoma growth, both absolute and proportional, will be studied where these data were collected by individual trials, and is based on central measurements from semi-automated planimetric[21] or ABC/2[22] methods; (ii) other imaging outcomes (e.g. perihaematomal oedema) will be included in supplemental analyses.

Subgroup analyses

Where adequate data are available, heterogeneity in the effect of BP lowering on outcomes will be assessed in the following subgroups to determine whether any effects of BP lowering are moderated by patient characteristics and BP lowering treatment (agent, target, timing, or place of delivery):

For peer review only

- Baseline characteristics: demographic (age, sex, region), clinical (stroke severity, baseline systolic BP, pre-stroke antihypertensive drug use, pre-stroke antithrombotic use), and radiological parameters (ICH volume, pre-stroke characteristics).
- BP lowering strategy: intervention/class-based treatment, BP target-based treatment.
- Timing of intervention: <2 hours, 2-6 hours, 6-48 hours, >48 hours after onset of ICH.
- Type of trial: pre-hospital vs. hospital, ICH only vs. mixed.

Associations of calculated BP parameters and primary outcome

Once all data have been pooled, an assessment of the new dataset will be made with a view to use trial BP and heart rate measures to calculate important BP lowering haemodynamic parameters, a number of which have been associated with outcome after ICH.[23–26] The aim is to determine the prognostic significance of these calculated variables, adjusted for all known confounders, and to present these data in secondary analyses.

Risk of bias in individual studies

Two investigators (TJM and ZKL) will assess each included study for bias using the Cochrane Collaboration tool (http://methods.cochrane.org/bias/assessing-risk-bias-included-studies). A judgement of bias will be made according to 6 domains: selection, performance, detection, attrition, reporting and 'other' biases. Disagreements will be resolved with discussion, or by involving PMB if necessary. Any uncertainties about trial design, conduct or analysis methods will be clarified with the individual trial's investigators.

Statistical analyses

Primary analyses will be performed using the intention-to-treat dataset from each trial with a one-stage approach. Patients without available data, or where the above procedure for missing data cannot be applied, will be excluded from these analyses.

Supplementary material 3 contains table and figure shells that will be used to present our findings. In summary, descriptive analyses will be undertaken in order to identify key similarities and differences between trials, therefore providing context for interpretation of between-trial differences in outcomes. Data will be described as mean (standard deviation) or median (interquartile range) for continuous data, or frequency (percentage) for categorical data, and chi-squared or Kruskal-Wallis tests will be used to make comparisons.

Generalised linear mixed models will be used with covariates (age, sex, ICH severity, time from onset to randomisation), and the source trial added as a random effect to account for

For peer review only - http://bmjopen.bmj.com/site/about/guidelines.xhtml

clustering. For completeness, analyses will also be performed unadjusted. Results from binary and ordinal analyses (e.g. death, dichotomised outcomes of mRS scale, neurologic deterioration, serious adverse events, full-scale mRS) will be presented as odds ratios (OR) with 95% confidence intervals (CI). The proportional odds assumption will be checked before ordinal analyses of outcomes on the mRS are undertaken. If the proportional odds assumption is not met, various standard binary cut-points for poor outcome on the mRS will be used. Continuous or pseudo-continuous outcome analysis results will be presented as mean difference (MD) with 95% CI. For time-to-event outcomes, a Cox proportional hazards model will be used to determine hazard ratios (HR) with the source trial added as a random effect, and the assumption of proportional hazards will be tested.

Additional analyses will be performed in pre-specified subgroups, with an interaction term in models to test heterogeneity.

Sensitivity analyses using a two-stage approach will be conducted to test the robustness of our primary results, and will permit: (1) inclusion of aggregate data from studies where IPD cannot be obtained to address the issue of data availability bias;[27] and (2) assessment with and without studies deemed to have high risk of bias.

An assessment of heterogeneity will be performed before data pooling using the Cochrane Q statistic and I^2 statistic.

An assessment of publication bias will be made by visual inspection for funnel plot asymmetry (with and without studies where IPD is obtained) and with Egger's regression test. The Grades of Recommendation, Assessment, Development, and Evaluation (GRADE)[28] will be used to evaluate the quality of the synthesised evidence.

Ethics and dissemination

Ethical approval for the original studies was sought and is documented elsewhere. No new patient data will be collected nor is there any deviation from the original purposes of each study, therefore further ethical approval is not required. Results from this study will be published in international peer-reviewed journals. All publications from this work will be in name of the BASC Investigators.

Discussion

The proposed study will use pooled IPD from RCTs to address the role of BP management in the acute phase of ICH. Conflicting results from multiple clinical trials indicate that BP

For peer review only - http://bmjopen.bmj.com/site/about/guidelines.xhtml
For peer review only

reduction is not a straightforward question of whether to treat or not. Rather, the problem is complex and needs to take account of patient characteristics, physiological parameters including baseline BP, stroke severity, the timing of treatment, strategy and class of antihypertensive agent, route of administration, and dose or target BP.

Elevated baseline systolic BP is associated with haematoma expansion,[29] perihaematomal oedema formation,[30] and increased case fatality.[31] There is evidence from some studies that intensive lowering of BP reduces haematoma enlargement,[32] is safe and tolerable,[2,3] and does not alter cerebral blood flow.[33] Current guidelines recommend lowering BP early in the course after ICH and that targeting a goal of systolic BP <140 mmHg is probably safe in patients presenting with an SBP of 150 to 220 mm Hg.[34,35] Nevertheless, other studies reported no effect of BP reduction on haematoma enlargement,[3,4] and the neutral findings of the ATACH-II trial[4] emphasise that all published data need to be appraised together.

Although guidelines recognise the need for very early treatment, none address the role of pre-hospital BP reduction. Further evidence is also required to confirm associations between key haemodynamic variables, such as BP variability,[23] with poor outcome. Furthermore, the influence of patient characteristics that are present prior to ICH on BP lowering interventions is unknown, although these same variables are known to worsen long-term recovery; these include having a prior stroke, white matter lesions or brain atrophy. The proposed investigation aims to provide definitive evidence on these important issues.

Acknowledgements

The protocol paper is written on behalf of the Blood pressure in Acute Stroke Collaboration (BASC, convener PMB). BASC-ICH comprises:

ATACH-II: Adnan I Qureshi, Yuko Palesch

CHHIPS: John Potter

ENOS: Philip M Bath, Nikola Sprigg, Joanna M Wardlaw

FAST-Mag: Jeff Saver, Nerses Sanossian

GTN-1/2/3, RIGHT: Philip M Bath

ICH-ADAPT: Ken Butcher

IMAGES: Kennedy R Lees, Keith W Muir

INTERACT: Craig S Anderson

For peer review only - http://bmjopen.bmj.com/site/about/guidelines.xhtml

INTERACT2: Craig S Anderson, Hisatomi Arima

PILFAST: Gary Ford

RIGHT-2: Philip M Bath, Nikola Sprigg, Joanna M Wardlaw

SCAST: Eivind Berge, Else C Sandset

VENUS: Janneke Horn

Author contributions

PMB, TJM, ECS and XW were responsible for first draft of the protocol, study conception and critical review of the current protocol. XW and LJW were responsible for statistical oversight. ZKL, CD, NS, KK, TGR, JW, RASS, EB and CSA were responsible for study conception and critical review of the current protocol.

Funding statement

BASC has not received specific grants from any funding agency in the public, commercial, or not-for-profit sectors. Potential trials for inclusion have reported their own funding. TJM is a British Heart Foundation clinical research training fellow. PMB is a Stroke Association Professor of Stroke Medicine. PMB and TGR are NIHR Senior Investigators. CSA is a Senior Research Fellow of the National Health and Medical Research Council (NHMRC) of Australia.

Competing interests statement

None declared

Data statement

BASC is performing protocol-driven IPD meta-analyses of trial data shared by investigators. It is not a data repository and requests for the source data should be made to the relevant chief investigator.

For peer review only

References

1 Bath PMW, Krishnan K. Interventions for deliberately altering blood pressure in acute stroke. *Cochrane database Syst Rev* 2014;**10**:CD000039.

2 Anderson CS, Huang Y, Wang JG, *et al.* Intensive blood pressure reduction in acute cerebral haemorrhage trial (INTERACT): a randomised pilot trial. *Lancet Neurol* 2008;**7**:391–9.

3 Anderson C, Heeley E, Huang Y, *et al.* Rapid blood-pressure lowering in patients with acute intracerebral hemorrhage. *N Engl J Med* 2013;**368**:2355–65.

4 Qureshi AI, Palesch YY, Barsan WG, *et al.* Intensive blood pressure lowering in patients with acute cerebral hemorrhage. *N Engl J Med* 2016;**375**:1033–43.

5 Saver JL, Starkman S, Eckstein M, *et al.* Prehospital use of magnesium sulfate as neuroprotection in acute Stroke. *N Engl J Med* 2015;**372**:528–36.

6 Shaw L, Price C, McLure S, *et al.* Paramedic initiated lisinopril for acute stroke treatment (PIL-FAST): Study protocol for a pilot randomised controlled trial. *Trials* 2011;**12**:152.

7 Ankolekar S, Fuller M, Cross I, *et al.* Feasibility of an ambulance-based stroke trial, and safety of glyceryl trinitrate in ultra-acute stroke: The rapid intervention with glyceryl trinitrate in hypertensive stroke trial. *Stroke* 2013;**44**:3120–8.

8 Sandset EC, Bath PMW, Boysen G, *et al.* The angiotensin-receptor blocker candesartan for treatment of acute stroke (SCAST): A randomised, placebo-controlled, double-blind trial. *Lancet* 2011;**377**:741–50.

9 Bath PMW, Woodhouse L, Scutt P, *et al.* Efficacy of nitric oxide, with or without continuing antihypertensive treatment, for management of high blood pressure in acute stroke (ENOS): a partial-factorial randomised controlled trial. *Lancet* 2015;**385**:617–28.

10 Potter JF, Robinson TG, Ford GA, *et al.* Controlling hypertension and hypotension immediately post-stroke (CHHIPS): a randomised, placebo-controlled, double-blind pilot trial. *Lancet Neurol* 2009;**8**:48–56.

11 Robinson TG, Potter JF, Ford GA, *et al.* Effects of antihypertensive treatment after acute stroke in the continue or stop post-stroke antihypertensives collaborative study

For peer review only - http://bmjopen.bmj.com/site/about/guidelines.xhtml

(COSSACS): a prospective, randomised, open, blinded-endpoint trial. *Lancet Neurol* 2010;**9**:767–75.

12 Koch S, Romano JG, Forteza AM, *et al.* Rapid blood pressure reduction in acute intracerebral hemorrhage: Feasibility and safety. *Neurocrit Care* 2008;**8**:316–21.

13 Bath PM, Appleton JP, Krishnan K, *et al*. Blood pressure in acute stroke: to treat or not to treat: that is still the question. *Stroke* 2018;**49**:1784–90.

14 Bath FJ, Bath PMW. What is the correct management of blood pressure in acute stroke? The blood pressure in acute stroke collaboration. *Cerebrovasc Dis* 1997;**7**:205–13.

15 Sandset EC, Sanossian N, Woodhouse LJ, *et al.* Protocol for a prospective collaborative systematic review and meta-analysis of individual patient data from randomized controlled trials of vasoactive drugs in acute stroke: the blood pressure in acute stroke collaboration, stage-3. *Int J Stroke* 2018; 1:1747493018772733.

16 Clarke MJ, Stewart LA. Systematic Reviews: Obtaining data from randomised controlled trials: how much do we need for reliable and informative meta-analyses? *BMJ* 1994;**309**:1007.

17 Lattanzi S, Cagnetti C, Provinciali L *et al*. How should ee lower blood pressure after cerebral hemorrhage? a systematic review and meta-analysis. *Cerebrovasc Dis* 2017;**43**:207-13

18 Tierney JF, Vale C, Riley R, *et al.* Individual participant data (IPD) meta-analyses of randomised controlled trials: guidance on their use. *PLoS Med* 2015;**12**:e1001855.

19 Stewart LA, Clarke M, Rovers M, *et al.* Preferred reporting items for systematic review and meta-analyses of individual participant data: the PRISMA-IPD statement. *JAMA* 2015;**313**:1657–65.

20 Booth A, Clarke M, Dooley G, *et al.* The nuts and bolts of PROSPERO: An international prospective register of systematic reviews. *Syst Rev* 2012;**1**.

21 Bardera A, Boada I, Feixas M, *et al.* Semi-automated method for brain hematoma and edema quantification using computed tomography. *Comput Med Imaging Graph* 2009;**33**:304–11.

22 Kothari RU, Brott T, Broderick JP, *et al.* The ABCs of measuring intracerebral

For peer review only - http://bmjopen.bmj.com/site/about/guidelines.xhtml

hemorrhage volumes. *Stroke* 1996;**27**:1304–5.

23 Manning L, Hirakawa Y, Arima H, *et al.* Blood pressure variability and outcome after acute intracerebral haemorrhage: A post-hoc analysis of INTERACT2, a randomised controlled trial. *Lancet Neurol* 2014;**13**:364–73.

24 Wang X, Arima H, Heeley E, *et al.* Magnitude of blood pressure reduction and clinical outcomes in acute intracerebral hemorrhage: Intensive blood pressure reduction in acute cerebral hemorrhage trial study. *Hypertension* 2015;**65**:1026–32.

25 Carcel C, Wang X, Sato S, *et al.* Degree and timing of intensive blood pressure lowering on hematoma growth in intracerebral hemorrhage: intensive blood pressure reduction in acute cerebral hemorrhage trial-2 results. *Stroke* 2016;**47**:1651–3.

26 Arima H, Heeley E, Delcourt C, *et al.* Optimal achieved blood pressure in acute intracerebral hemorrhage: INTERACT2. *Neurology* 2015;**84**:464–71.

27 Ahmed I, Sutton AJ, Riley RD. Assessment of publication bias, selection bias, and unavailable data in meta-analyses using individual participant data: A database survey. *BMJ* 2012;**344**.

28 Iorio A, Spencer FA, Falavigna M, *et al.* Use of GRADE for assessment of evidence about prognosis: rating confidence in estimates of event rates in broad categories of patients. *BMJ* 2015;**350**.

29 Kazui S, Minematsu K, Yamamoto H, *et al.* Predisposing factors to enlargement of spontaneous intracerebral hematoma. *Stroke* 1997;**28**:2370–5.

30 Vemmos KN, Tsivgoulis G, Spengos K, *et al.* Association between 24-h blood pressure monitoring variables and brain oedema in patients with hyperacute stroke. *J Hypertens* 2003;**21**:2167–73.

31 Zhang Y, Reilly KH, Tong W, *et al.* Blood pressure and clinical outcome among patients with acute stroke in inner Mongolia, China. *J Hypertens* 2008;**26**:1446–52.

32 Anderson CS, Huang Y, Arima H, *et al.* Effects of early intensive blood pressure-lowering treatment on the growth of hematoma and perihematomal edema in acute intracerebral hemorrhage: the intensive blood pressure reduction in acute cerebral haemorrhage trial (INTERACT). *Stroke* 2010;**41**:307–12.

33 Butcher K, Jeerakathil T, Emery D, *et al.* The intracerebral haemorrhage acutely

For peer review only

For peer review only - http://bmjopen.bmj.com/site/about/guidelines.xhtml

decreasing arterial pressure trial: ICH ADAPT. *Int J Stroke* 2010;**5**:227–33.

34 Steiner T, Al-Shahi Salman R, Beer R, *et al.* European Stroke Organisation (ESO) guidelines for the management of spontaneous intracerebral hemorrhage. *Int J Stroke* 2014;**9**:840–55.

35 Hemphill JC, Greenberg SM, Anderson CS, *et al.* Guidelines for the management of spontaneous intracerebral hemorrhage. *Stroke* 2015;**46**:2032–60.

For peer review only

For peer review only - http://bmjopen.bmj.com/site/about/guidelines.xhtml

SUPPLEMENTARY MATERIAL 1: Search Strategy

Medline search strategy

1. exp basal ganglia hemorrhage/ or intracranial hemorrhages/ or cerebral hemorrhage/ or intracranial hemorrhage, hypertensive/ or cerebrovascular disorders/

2. ((brain$ or cerebr$ or cerebell$ or intracerebral or intracran$ or parenchymal or intraparenchymal or intraventricular or infratentorial or supratentorial or basal gangli$ or putaminal or putamen or posterior fossa or hemispher$ or pon$ or lentiform$ or brainstem or cortic$ or cortex$ or subcortic$ or subcortex$) adj5 (h?emorrhag$ or h?ematoma$ or bleed$)).tw

3. ((hemorrhag$ or haemorrhag$) adj6 (stroke$ or apoplex$ or cerebral vasc$ or cerebrovasc$ or cva)).tw

4. (ICH or ICHs or PICH or PICHs).tw

5. 1 or 2 or 3 or 4

6. exp blood pressure/

7. exp hypertension/

8. (blood pressure or bloodpressure).tw

9. ((bp or blood pressure) adj5 (lowering or reduc$)).tw

10. ((strict$ or target$ or tight$ or intens$ or below) adj3 (blood pressure or systolic or diastolic or bp or level$)).tw

11. (hypertension or hypertensive).tw

12. ((manage$ or monitor$) adj3 (hypertension or blood pressure)).tw

13. ((intense or intensive or aggressive or accelerated or profound or radical or severe) adj5 ((bp or blood pressure) adj5 (lowering or reduc$ or decreas$ or decrement or dimin$ or declin$))).tw

14. ((standard or normal or ordinary or guideline or guide line or guideline recommend$ or recommend$ or convention$ or usual or established) adj5 ((bp or blood pressure) adj5 (lowering or reduc$ or decreas$ or decrement or dimin$ or declin$))).tw

15. (antihypertensive adj2 (agent$ or drug$ or medicat$)).tw

For peer review only

For peer review only - http://bmjopen.bmj.com/site/about/guidelines.xhtml

16. 6 or 7 or 8 or 9 or 10 or 11 or 12 or 13 or 14 or 15

17. exp nitroglycerin/

18. nitrate.tw

19. glyceryl trinitrate.mp. or GTN.tw. [mp=title, abstract, original title, name of substance word, subject heading word, keyword heading word, protocol supplementary concept word, rare disease supplementary concept word, unique identifier, synonyms]

20. nitric Oxide Donors.tw

21. exp angiotensin-converting enzyme inhibitor/

22. exp ace inhibitor/

23. angiotensin-converting enzyme inhibitor$.mp

24. ace inhibitor$.mp

25. (alacepril or altiopril or benazepril or captopril or ceronapril or cilazapril or delapril or derapril or enalapril or fosinopril or idapril or imidapril or lisinopril or moexipril or moveltipril or pentopril or perindopril or quinapril or ramipril or spirapril or temocapril or randolapril or zofenopril).mp

26. exp Angiotensin Receptor Antagonists/

27. (angiotensin adj3 (receptor antagon$ or receptor block$)).tw

28. arb?.tw

29. (abitesartan or azilsartan or candesartan or elisartan or embusartan or eprosartan or forasartan or irbesartan or losartan or milfasartan or olmesartan or saprisartan or tasosartan or telmisartan or valsartan or zolasartan).mp

30. exp adrenergic beta-antagonists/

31. (beta adj2 (adrenergic? or antagonist? or block$ or receptor?)).tw

32. (acebutolol or alprenolol or amosulalol or arotinolol or atenolol or befunolol or betaxolol or bevantolol or bisoprolol or bopindolol or bucindolol or bucumolol or bufetolol or bufuralol or bunitrolol or bupranolol or butofilolol or carazolol or carteolol or carvedilol or celiprolol or cetamolol or cloranolol or cyanopindolol or deacetylmetipranolol or dihydroalprenolol or dilevalol or epanolol or esmolol or indenolol or iodocyanopindolol or labetalol or landiolol or levobunolol or mepindolol or metoprolol or metipranolol or moprolol or nadolol or nadoxolol

For peer review only - http://bmjopen.bmj.com/site/about/guidelines.xhtml

or nebivolol or nifenalol or nipradilol or oxprenolol or penbutolol or pindolol or practolol or pronethalol or propranolol or proxodolol or sotalol or sulfinalol or talinolol or tertatolol or tilisolol or timolol or toliprolol or xibenolol).mp

33. exp calcium channel blockers/

34. (calcium adj2 (inhibit$ or agonist? or exogenous or blockader?)).tw

35. (calcium channel blockers or amlodipine or amrinone or bencyclane or bepridil or cinnarizine or conotoxins or diltiazem or felodipine or fendiline or flunarizine or gallopamil or isradipine or lidoflazine or magnesium sulfate or mibefradil or nicardipine or nifedipine or nimodipine or nisoldipine or nitrendipine or perhexiline or prenylamine or verapamil or omega-agatoxin iva or omega-conotoxin gvia or omega-conotoxins).mp

36. exp diuretics/

37. (thiazide or furosemide or bumetanide or piretanide or torasemide or azosemide or ethacrynic acid or ticrynafen or trip amide or phenoxybenzoic acid or muzolimine or indacrinone or etozolin or ozolinone or cicletanine or cicletanine or tienilic acid or tizolemide or hydrochlorothiazide or chlorothiazide or buthiazide or bendroflumethiazide or hydroflumethiazide or trichlormethiazide or methylclothiazide or polythiazide or cyclothiazide or cyclopenthiazide or chlorthalidone or metolazone or quinethazone or fenquizone or clorexolone or clop amide or indapamide or diapamide or isodapamide or mefruside or xipamide or diuretic$).mp

38. exp adrenergic alpha antagonists/

39. (alpha adrenergic antagonist? or alpha adrenergic receptor antagonist? or adrenergic alpha antagonist?).tw

40. ((alpha or alpha-adrenergic) adj2 block$).tw

41. (alfuzosin or bunazosin or doxazosin or indoramin or metazosin or neldazosin or prazosin or silodosin or tamsulosin or terazosin or tiodazosin or trimazosin or urapidil or phentolamine or phenoxybenzamine).mp

42. (methyldopa or alphamethyldopa or amodopa or dopamet or dopegyt or dopegit or dopegite or emdopa or hyperpax or hyperpaxa or methylpropionic acid or dopergit or meldopa or methyldopate or medopa or medomet or sembrina or aldomet or aldometil or aldomin or hydopa or methyldihydroxyphenylalanine or methyl dopa or mulfasin or presinol

For peer review only - http://bmjopen.bmj.com/site/about/guidelines.xhtml

or presolisin or sedometil or sembrina or taquinil or dihydroxyphenylalanine or methylphenylalanine or methylalanine or alpha methyl dopa).mp.

43. (alazine or alphapress or apdormin or apresolin or apresolina or apresoline or apressin or apressoline or aprezin or clorana or depressan or deselazin or dihydralazine or dihydrallazine or dihydrazinophthalazin or dralzine or hidral or hydralacin or hydralazine or hydralazine or hydralizine or hydrallazin or hydralazine or hydrapres or hydrazinophtalizine or hydrazinophthalazine or hydrolazine or hypatol or hyperphen or hypoftalin or hypophthalin or idralazina or iopress or nepresol or nonpolin or novo-hylazin or plethorit or praeparat or resporidin or solesorin or tetrasoline or travinon).mp

44. exp nitroprusside/

45. aliskiren.mp

46. renin inhibitor$.mp

47. moxonidine.mp

48. (minoxidil or minoxidine).mp. [mp=title, abstract, original title, name of substance word, subject heading word, keyword heading word, protocol supplementary concept word, rare disease supplementary concept word, unique identifier, synonyms]

49. clonidine.mp

50. guanfacine.mp

51. 17 or 18 or 19 or 20 or 21 or 22 or 23 or 24 or 25 or 26 or 27 or 28 or 29 or 30 or 31 or 32 or 33 or 34 or 35 or 36 or 37 or 38 or 39 or 40 or 41 or 42 or 43 or 44 or 45 or 46 or 47 or 48 or 49 or 50

52. 16 or 51

53. randomized controlled trial/

54. randomization/

55. Clinical Trial/

56. (clin$ adj25 trial$).ti,ab

57. ((singl$ or doubl$ or trebl$ or tripl$) adj25 (blind$ or mask$)).ti,ab

58. placebo$.ti,ab

59. random$.ti,ab

For peer review only - http://bmjopen.bmj.com/site/about/guidelines.xhtml

60. 53 or 54 or 55 or 56 or 57 or 58 or 59

61. exp animals/ not humans.sh.

62. 60 not 61

63. 5 and 52 and 62

For peer review only

For peer review only - http://bmjopen.bmj.com/site/about/guidelines.xhtml

Embase search strategy

1. exp basal ganglia hemorrhage/ or intracranial hemorrhages/ or cerebral hemorrhage/ or intracranial hemorrhage, hypertensive/ or cerebrovascular disorders/

2. ((brain$ or cerebr$ or cerebell$ or intracerebral or intracran$ or parenchymal or intraparenchymal or intraventricular or infratentorial or supratentorial or basal gangli$ or putaminal or putamen or posterior fossa or hemispher$ or pon$ or lentiform$ or brainstem or cortic$ or cortex$ or subcortic$ or subcortex$) adj3 (h? emorrhag$ or h?ematoma$ or bleed$)).tw

3. ((hemorrhag$ or haemorrhag$) adj3 (stroke$ or apoplex$ or cerebral vasc$ or cerebrovasc$ or cva)).tw

4. (ICH or ICHs or PICH or PICHs).tw

5. 1 or 2 or 3 or 4

6. exp blood pressure/

7. exp hypertension/

8. (blood pressure or bloodpressure).tw

9. (hypertension or hypertensive).tw

10. ((bp or blood pressure) adj3 (lowering or reduc$)).tw

11. ((strict$ or target$ or tight$ or intens$ or below) adj3 (blood pressure or systolic or diastolic or bp or level$)).tw

12. ((manage$ or monitor$) adj3 (hypertension or blood pressure)).tw

13. ((intense or intensive or aggressive or accelerated or profound or radical or severe) adj3 ((bp or blood pressure) adj3 (lowering or reduc$ or decreas$ or decrement or dimin$ or declin$))).tw

14. ((standard or normal or ordinary or guideline or guide line or guideline recommend$ or recommend$ or convention$ or usual or established) adj3 ((bp or blood pressure) adj3 (lowering or reduc$ or decreas$ or decrement or dimin$ or declin$))).tw

15. (antihypertensive adj2 (agent$ or drug$ or medicat$)).tw

16. exp antihypertensive agents/

17. exp antihypertensive therapy/

For peer review only - http://bmjopen.bmj.com/site/about/guidelines.xhtml

18. 6 or 7 or 8 or 9 or 10 or 11 or 12 or 13 or 14 or 15 or 16 or 17

19. exp glyceryl trinitrate/

20. nitrate.tw

21. glyceryl trinitrate.mp. or GTN.tw

22. nitric Oxide Donors.tw

23. exp peptidyl carboxypeptidase inhibitor/

24. exp ace inhibitor/

25. angiotensin-converting enzyme inhibitor$.mp

26. ace inhibitor$.mp

27. (alacepril or altiopril or benazepril or captopril or ceronapril or cilazapril or delapril or derapril or enalapril or fosinopril or idapril or imidapril or lisinopril or moexipril or moveltipril or pentopril or perindopril or quinapril or ramipril or spirapril or temocapril or trandolapril or zofenopril).mp

28. exp Angiotensin Receptor Antagonists/

29. (angiotensin adj3 (receptor antagon$ or receptor block$)).tw

30. arb?.tw

31. (abitesartan or azilsartan or candesartan or elisartan or embusartan or eprosartan or forasartan or irbesartan or losartan or milfasartan or olmesartan or saprisartan or tasosartan or telmisartan or valsartan or zolasartan).mp

32. exp beta adrenergic receptor blocking agent/

33. (beta adj2 (adrenergic? or antagonist? or block$ or receptor?)).tw

34. (acebutolol or alprenolol or amosulalol or arotinolol or atenolol or befunolol or betaxolol or bevantolol or bisoprolol or bopindolol or bucindolol or bucumolol or bufetolol or bufuralol or bunitrolol or bupranolol or butofilolol or carazolol or carteolol or carvedilol or celiprolol or cetamolol or cloranolol or cyanopindolol or deacetylmetipranolol or dihydroalprenolol or dilevalol or epanolol or esmolol or indenolol or iodocyanopindolol or labetalol or landiolol or levobunolol or mepindolol or metoprolol or metipranolol or moprolol or nadolol or nadoxolol or nebivolol or nifenalol or nipradilol or oxprenolol or penbutolol or pindolol or practolol or

For peer review only - http://bmjopen.bmj.com/site/about/guidelines.xhtml

pronethalol or propranolol or proxodolol or sotalol or sulfinalol or talinolol or tertatolol or tilisolol or timolol or toliprolol or xibenolol).mp

35. exp calcium channel blocking agent/

36 (calcium adj2 (inhibit$ or agonist? or exogenous or blockader?)).tw

37. (calcium channel blockers or amlodipine or amrinone or bencyclane or bepridil or cinnarizine or conotoxins or diltiazem or felodipine or fendiline or flunarizine or gallopamil or isradipine or lidoflazine or magnesium sulfate or mibefradil or nicardipine or nifedipine or nimodipine or nisoldipine or nitrendipine or perhexiline or prenylamine or verapamil or omega-agatoxin iva or omegaconotoxin gvia or omega-conotoxins).mp

38. exp diuretic agent/

39. (thiazide or furosemide or bumetanide or piretanide or torasemide or azosemide or ethacrynic acid or ticrynafen or trip amide or phenoxybenzoic acid or muzolimine or indacrinone or etozolin or ozolinone or cicletanine or cicletanine or tienilic acid or tizolemide or hydrochlorothiazide or chlorothiazide or buthiazide or bendroflumethiazide or hydroflumethiazide or trichlormethiazide or methylclothiazide or polythiazide or cyclothiazide or cyclopenthiazide or chlorthalidone or metolazone or quinethazone or fenquizone or clorexolone or clop amide or indapamide or diapamide or isodapamide or mefruside or xipamide or diuretic$).mp

40. exp alpha adrenergic receptor blocking agent/

41. (alpha adrenergic antagonist? or alpha adrenergic receptor antagonist? or adrenergic alpha antagonist?).tw

42. ((alpha or alpha-adrenergic) adj2 block$).tw

43. (alfuzosin or bunazosin or doxazosin or indoramin or metazosin or neldazosin or prazosin or silodosin or tamsulosin or terazosin or tiodazosin or trimazosin or urapidil or phentolamine or phenoxybenzamine).mp

44. (methyldopa or alphamethyldopa or amodopa or dopamet or dopegyt or dopegit or dopegite or emdopa or hyperpax or hyperpaxa or methylpropionic acid or dopergit or meldopa or methyldopate or medopa or medomet or sembrina or aldomet or aldometil or aldomin or hydopa or methyldihydroxyphenylalanine or methyl dopa or mulfasin or presinol or presolisin or sedometil or sembrina or taquinil or dihydroxyphenylalanine or methylphenylalanine or methylalanine or alpha methyl dopa).mp

For peer review only

45. (alazine or alphapress or apdormin or apresolin or apresolina or apresoline or apressin or apressoline or aprezin or clorana or depressan or deselazin or dihydralazine or dihydrallazine or dihydrazinophthalazin or dralzine or hidral or hydralacin or hydralazine or hydralazine or hydralizine or hydrallazin or hydralazine or hydrapres or hydrazinophtalizine or hydrazinophthalazine or hydrolazine or hypatol or hyperphen or hypoftalin or hypophthalin or idralazina or iopress or nepresol or nonpolin or novo-hylazin or plethorit or praeparat or resporidin or solesorin or tetrasoline or travinon).mp

46. exp nitroprusside sodium/

47. aliskiren.mp

48. renin inhibitor$.mp

49. moxonidine.mp

50. (minoxidil or minoxidine).mp. [mp=title, abstract, original title, name of substance word, subject heading word, keyword heading word, protocol supplementary concept word, rare disease supplementary concept word, unique identifier] {Including Related Terms}

51. clonidine.mp

52. guanfacine.mp

53. 19 or 20 or 21 or 22 or 23 or 24 or 25 or 26 or 27 or 28 or 29 or 30 or 31 or 32 or 33 or 34 or 35 or 36 or 37 or 38 or 39 or 40 or 41 or 42 or 43 or 44 or 45 or 46 or 47 or 48 or 49 or 50 or 51 or 52

54. 18 or 53

55. crossover-procedure.sh

56. double-blind procedure.sh

57. single-blind procedure.sh

58. (random$ or factorial$ or crossover$ or cross over$ or cross-over$ or placebo$ or (doubl$ adj blind$) or (singl$ adj blind$) or assign$ or allocat$).tw,ot

59. randomized controlled trial.sh

60. trial.ti

61. controlled clinical trial/

62. 55 or 56 or 57 or 58 or 59 or 60 or 61

For peer review only - http://bmjopen.bmj.com/site/about/guidelines.xhtml

63. exp animal/ or exp invertebrate/ or animal.hw. or non human/ or nonhuman/

64. human/ or human cell/ or human tissue/ or normal human/

65. 63 not 64

66. 62 not 65

For peer review only

For peer review only - http://bmjopen.bmj.com/site/about/guidelines.xhtml

Cochrane Central Register of Controlled Trials

#1 MeSH descriptor: [Basal Ganglia Hemorrhage] explode all trees

#2 MeSH descriptor: [Intracranial Hemorrhage, Hypertensive] explode all trees

#3 MeSH descriptor: [Cerebral Hemorrhage] explode all trees

#4 MeSH descriptor: [Cerebrovascular Disorders] this term only

#5 (hemorrhag* or haemorrhag*) ti.ab.kw

#6 (stroke* or apoplex* or cerebral vasc*? or cerebrovasc* or cva) ti.ab.kw

#7 #5 and #6

#8 (brain* or cerebr* or cerebell* or intracerebral or intracran* or parenchymal or infratentorial or supratentorial or basal gangli*? or putaminal or putamen or posterior fossa? or pons or pontine or brainstem or lentiform) ti.ab.kw

#9 (haemorrhage* or hemorrhage* or haematoma* or hematoma* or bleed*) ti.ab.kw

#10 #8 and #9

#11 #1 or #2 or #3 or #4 or #7 or #10

#12 ((bp or blood pressure) adj5 (lowering or reduc$)) .tw

#13 ((strict$ or target$ or tight$ or intens$ or below) adj3 (blood pressure or systolic or diastolic or bp or level$)) .tw

#14 (hypertension or hypertensive) .tw

#15 ((manage$ or monitor$) adj3 (hypertension or blood pressure)) .tw

#16 ((intense or intensive or aggressive or accelerated or profound or radical or severe) adj5 ((bp or blood pressure) adj5 (lowering or reduc$ or decreas$ or decrement or dimin$ or declin $))) .tw

#17 ((standard or normal or ordinary or guideline or guide line or guideline recommend$ or recommend$ or convention$ or usual or established) adj5 ((bp or blood pressure) adj5 (lowering or reduc$ or decreas$ or decrement or dimin$ or declin$))) .tw

#18 (antihypertensive adj2 (agent$ or drug$ or medicat$)) .tw

#19 MeSH descriptor: [Antihypertensive Agents] explode all trees

#20 #12 or #13 or #14 or #15 or #16 or #17 or #18 or #19

For peer review only - http://bmjopen.bmj.com/site/about/guidelines.xhtml

#21 MeSH descriptor: [Angiotensin-Converting Enzyme Inhibitors] explode all trees

#22 angiotensin-converting enzyme inhibitor$.mp

#23 ace inhibitor$.mp

#24 (alacepril or altiopril or benazepril or captopril or ceronapril or cilazapril or delapril or derapril or enalapril or fosinopril or idapril or imidapril or lisinopril or moexipril or moveltipril or pentopril or perindopril or quinapril or ramipril or spirapril or temocapril or trandolapril or zofenopril) .mp

#25 MeSH descriptor: [Angiotensin Receptor Antagonists] explode all trees 2059

#26 (angiotensin adj3 (receptor antagon$ or receptor block $)) .tw

#27 arb?.tw

#28 (abitesartan or azilsartan or candesartan or elisartan or embusartan or eprosartan or forasartan or irbesartan or losartan or milfasartan or olmesartan or saprisartan or tasosartan or telmisartan or valsartan or zolasartan) .mp

#29 MeSH descriptor: [Adrenergic beta-Antagonists] explode all trees 4548

#30 (beta adj2 (adrenergic? or antagonist? or block$ or receptor?)) .tw

#31 (acebutolol or alprenolol or amosulalol or arotinolol or atenolol or befunolol or betaxolol or bevantolol or bisoprolol or bopindolol or bucindolol or bucumolol or bufetolol or bufuralol or bunitrolol or bupranolol or butofilolol or carazolol or carteolol or carvedilol or celiprolol or cetamolol or cloranolol or cyanopindolol or deacetylmetipranolol or dihydroalprenolol or dilevalol or epanolol or esmolol or indenolol or iodocyanopindolol or labetalol or landiolol or levobunolol or mepindolol or metoprolol or metipranolol or moprolol or nadolol or nadoxolol or nebivolol or nifenalol or nipradilol or oxprenolol or penbutolol or pindolol or practolol or pronethalol or propranolol or proxodolol or sotalol or sulfinalol or talinolol or tertatolol or tilisolol or timolol or toliprolol or xibenolol) .mp

#32 MeSH descriptor: [Calcium Channel Blockers] explode all trees

#33 (calcium adj2 (inhibit$ or agonist? or exogenous or blockader?)) .tw

#34 (calcium channel blockers or amlodipine or amrinone or bencyclane or bepridil or cinnarizine or conotoxins or diltiazem or felodipine or fendiline or flunarizine or gallopamil or isradipine or lidoflazine or magnesium sulfate or mibefradil or nicardipine or nifedipine or

For peer review only - http://bmjopen.bmj.com/site/about/guidelines.xhtml

nimodipine or nisoldipine or nitrendipine or perhexiline or prenylamine or verapamil or omega-agatoxin iva or omegaconotoxin gvia or omega-conotoxins) .mp

#35 MeSH descriptor: [Diuretics] explode all trees

#36 (thiazide or furosemide or bumetanide or piretanide or torasemide or azosemide or ethacrynic acid or ticrynafen or trip amide or phenoxybenzoic acid or muzolimine or indacrinone or etozolin or ozolinone or cicletanine or cicletanine or tienilic acid or tizolemide or hydrochlorothiazide or chlorothiazide or buthiazide or bendroflumethiazide or hydroflumethiazide or trichlormethiazide or methylclothiazide or polythiazide or cyclothiazide or cyclopenthiazide or chlorthalidone or metolazone or quinethazone or fenquizone or clorexolone or clop amide or indapamide or diapamide or isodapamide or mefruside or xipamide or diuretic$) .mp

#37 MeSH descriptor: [Adrenergic alpha-Antagonists] explode all trees

#38 (alpha adrenergic antagonist? or alpha adrenergic receptor

antagonist? or adrenergic alpha antagonist?) .tw

#39 ((alpha or alpha-adrenergic) adj2 block$) .tw

#40 (alfuzosin or bunazosin or doxazosin or indoramin or metazosin or neldazosin or prazosin or silodosin or tamsulosin or terazosin or tiodazosin or trimazosin or urapidil or phentolamine or phenoxybenzamine) .mp

#41 (methyldopa or alphamethyldopa or amodopa or dopamet or dopegyt or dopegit or dopegite or emdopa or hyperpax or hyperpaxa or methylpropionic acid or dopergit or meldopa or methyldopate or medopa or medomet or sembrina or aldomet or aldometil or aldomin or hydopa or methyldihydroxyphenylalanine or methyl dopa or mulfasin or presinol or presolisin or sedometil or sembrina or taquinil or dihydroxyphenylalanine or methylphenylalanine or methylalanine or alpha methyl dopa) .mp

#42 (alazine or alphapress or apdormin or apresolin or apresolina or apresoline or apressin or apressoline or aprezin or clorana or depressan or deselazin or dihydralazine or dihydrallazine or dihydrazinophthalazin or dralzine or hidral or hydralacin or hydralazine or hydralazine or hydralizine or hydrallazin or hydralazine or hydrapres or hydrazinophtalizine or hydrazinophthalazine or hydrolazine or hypatol or hyperphen or hypoftalin or hypophthalin or idralazina or iopress or nepresol or nonpolin or novo-hylazin or plethorit or praeparat or resporidin or solesorin or tetrasoline or travinon) .mp

For peer review only - http://bmjopen.bmj.com/site/about/guidelines.xhtml

#43 MeSH descriptor: [Nitroprusside] explode all trees

#44 aliskiren.mp

#45 renin inhibitor$.mp

#46 moxonidine.mp

#47 (minoxidil or minoxidine) .mp

#48 clonidine.mp

#49 guanfacine.mp

#50 MeSH descriptor: [Nitroglycerin] explode all trees

#51 #21 or #22 or #23 or #24 or #25 or #26 or #27 or #28 or #29 or #30 or #31 or #32 or #33 or #34 or #35 or #36 or #37 or #38 or #39 or #40 or #41 or #42 or #43 or #44 or #45 or #46 or #47 or #48 or #49 or #50 17095

#52 #20 or #51 23976

#53 #11 and #52 306

For peer review only

SUPPLEMENTARY MATERIAL 2: Variables for extraction

Trial information	**Name of trial/acronym, chief investigator/study group, years conducted, number of participants randomised, number of participants treated (intervention vs. controls), number of participants with primary outcome.**
Demographics	**Age, sex,** ethnicity**, country**.
Medical history [Y/N]	Premorbid disability (mRS), **hypertension**, diabetes mellitus, ischaemic stroke, intracerebral haemorrhage, transient ischaemic attack, ischaemic heart disease including angina and myocardial infarction, congestive cardiac failure, atrial fibrillation, valvular heart disease, peripheral arterial disease, hyperlipidaemia, family history of young stroke, current alcohol excess, smoker (active/previous/never).
Medications at time of admission [Y/N]	**BP lowering therapy**: ACEi, ARB, BB, CCB, diuretic, AB, nitric oxide donor, centrally acting, other<br>**Anticoagulant therapy**: warfarin or non-VKA oral anticoagulant<br>**Antiplatelet therapy**: aspirin, clopidogrel, cilostazol, dipyridamole, other, two or more<br>Lipid lowering therapy: statin, other
Baseline clinical variables	**Systolic BP [mmHg], diastolic BP [mmHg], heart rate [beats per min],** presence of AF on electrocardiogram [Y/N], dysphagia [Y/N], **NIHSS score [/42], GCS score [/15]**, Scandinavian Neurological Stroke Scale [/58], stroke syndrome [TACS/PACS/LACS/POCS].
Baseline blood tests	eGFR [mL/min], creatinine [µmol/L or mg/dL], urea [mmol/L], sodium [mmol/L], potassium [mmol/L] blood glucose [mmol/L or mg/dL], osmolarity [calculated].
Baseline neuroimaging characteristics	**Scan availability [Y/N]**, CT [Y/N], CT-A [Y/N], MRI [Y/N], **ICH volume [mm^3 or mL], ICH location** [lobar or basal ganglia/deep or infratentorial/brainstem/cerebellar], **presence of intraventricular haemorrhage [Y/N]**, IVH volume [mm^3 or mL], CT-A spot sign [Y/N], mass effect [Y/N], perihaematomal oedema volume [mm^3 or mL], small vessel disease [Y/N], brain atrophy [Y/N], evidence of old stroke [Y/N].
BP treatment	**Time from onset to treatment [hours:minutes], treatment group [intensive vs. standard or intervention vs. control],** route of administration [IV/oral/transdermal/sublingual]

For peer review only - http://bmjopen.bmj.com/site/about/guidelines.xhtml

	IV antihypertensive agents: BB, CCB, diuretic, AB, nitric oxide donor, nitroprusside, hydralazine, other –name [Y/N]<br>Transdermal agents: GTN patch [Y/N]<br>Oral antihypertensive agent: ACEi, ARB, BB, CCB, diuretic, AB, central acting, other – name [Y/N]<br>Time to achieve target BP [hours:minutes]<br>**All trial BP and heart rate measurements within first 168 hours.**
Other management [Y/N]	Intubation, ICU/ITU, surgery [evacuation/ intraventricular drainage/ minimally invasive surgery/ other – name], VTE prophylaxis [heparin/ compression stockings/ intermittent pneumatic compression], mannitol, haemostatic agents, DNAR/withdrawal of active treatment.
Outcomes	**Death: within 90 days [Y/N]**<br>**Disability: mRS at day 7 and 90 [0-6],** NIHSS score at days 2, 3 and 7, Barthel index at day 7, 90, 180.<br>Quality of life: 5-level EQ5D score at 90 days [/1]<br>Cognitive function: MMSE at 90 days [/30]<br>**Serious adverse events: all fatal and non-fatal,** renal, VTE, pneumonia**, early neurologic deterioration, symptomatic hypotension requiring corrective therapy**<br>Haematoma characteristics on repeat brain imaging: ICH volume [mm^3 or mL], IVH volume [mm^3 or mL], perihaematomal oedema volume [mm^3 or mL]

Bold text indicates core variable

mRS indicates modified Rankin Scale; ACEi, angiotensin-converting enzyme inhibitor; ARB, angiotensin receptor blocker; BB, beta blocker; CCB, calcium channel blocker; AB, alpha blocker; BP, blood pressure; AF, atrial fibrillation; NIHSS, National Institute for Health Stroke Scale; GCS, Glasgow coma scale; TACS, total anterior circulation syndrome; PACS, partial anterior circulation syndrome; LACS, lacunar syndrome; POCS, posterior circulation syndrome; eGFR, estimated glomerular filtration rate; CT, computed tomography; CT-A, computed tomographic angiography; MRI, magnetic resonance imaging; ICH, intracerebral haemorrhage; IV, intravenous; GTN, glyceryl trinitrate; ICU/ITU, intensive care/treatment unit; VTE, venous thromboembolism; DNAR, do not attempt resuscitation, EQ-5D EuroQOL five dimensions health-related quality of life questionnaire

For peer review only - http://bmjopen.bmj.com/site/about/guidelines.xhtml

SUPPLEMENTARY MATERIAL 3: Table shells

TABLE 1. Baseline characteristics according to treatment group. Data are number (%), mean (standard deviation) or median (interquartile range), chi-squared or Kruskal-Wallis tests will be used to make comparisons.

	Active	Control	P
Age (years)			
Sex, female			
Geographical region			
North America			
Asia			
Europe			
Other			
ICH onset to randomisation (hrs)			
Medical history			
Hypertension			
Diabetes mellitus			
Stroke			
Ischaemic heart disease			
AF (history or ECG finding)			
Smoker			
Medication history			
Antihypertensive			
Antiplatelet			
Anticoagulation			
Clinical			
Systolic BP (mmHg)			
Diastolic BP (mmHg)			
Pulse pressure (mmHg)			
Heart rate (bpm)			

For peer review only

For peer review only - http://bmjopen.bmj.com/site/about/guidelines.xhtml

Rate-pressure product[1]	
NIHSS (/42)	
GCS (/15)	
Neuroimaging	
Haematoma volume (ml)	
Haematoma location	
Lobar	
Basal ganglia/deep	
Infratentorial	
Intraventricular haemorrhage	
Perihaematomal oedema	
Absolute (ml)	
Relative[2]	
Pre-stroke characteristics	
Leukoaraiosis	
Atrophy	
Prior stroke lesion	

ICH denotes intracerebral haemorrhage; AF, atrial fibrillation; BP, blood pressure; NIHSS, National Institute for Health stroke scale; GCS, Glasgow come scale

[1]Systolic BP multiplied by heart rate

[2]Perihaematomal oedema to intraparenchymal haemorrhage volume ratio

For peer review only

TABLE 2. Treatment used to lower blood pressure, and other processes of care. Data are number (%). Chi squared tests will be used to make comparisons.

	Treatment	Control	P
Route of administration			
Oral			
Intravenous			
Transdermal			
Sublingual			
BP intervention ^			
Target *			
ACE-I, po/sl §			
ARA, po +			
ß-RA, po/im §§			
CCB, iv ¥			
CCB, po $			
Nitrate, topical #			
Magnesium, iv ∫			
Other treatments (%)			
Mannitol			
Haemostatic agents			
VTE prophylaxis			
DNAR order/withdrawal of active treatment			
ITU/intubation			
Neurosurgery			

BP denotes blood pressure; ACE-I, angiotensin converting enzyme inhibitor; ARA, angiotensin receptor antagonist; ß-RA, beta receptor antagonist; CCB, calcium channel blocker; VTE, venous thromboembolism; DNAR, do not attempt resuscitation; ITU, intensive treatment unit; po, oral; sl, sublingual; im, intramuscular; iv, intravenous

^ Categorised according to current BASC collaborator trials

*INTERACT1&2, ATACH-II, ICH-ADAPT; § CHHIPS, ACEi arm; + SCAST; §§ CHHIPS, ß-RA arm; ¥ ATACH-II, Uzuner; $ VENUS; # GTN1&2, ENOS, RIGHT; ∫ IMAGES, FAST-Mag

For peer review only - http://bmjopen.bmj.com/site/about/guidelines.xhtml

TABLE 3. Effect of BP lowering on outcomes. Data are number (%), mean (standard deviation) or median (interquartile range); mean difference (MD) or odds ratios (OR) with 95% confidence intervals (CI). Generalised linear mixed models for binary, continuous and ordinal outcomes, with random intercept for source trial. Multivariable analyses adjusted for age, sex, severity (NIHSS), systolic BP, ICH volume, presence of IVH and time to randomisation.

	N	Treatment	Control	OR/MD	95% CI	P	OR/MD	95% CI	P
				Unadjusted			Adjusted		
**On treatment outcomes**									
Within 1 hour of randomisation									
Systolic BP (mmHg)									
Mean[1]				MD			MD		
Delta[2]				MD			MD		
Peak[3]				MD			MD		
Standard deviation[4]				MD			MD		
Diastolic BP § (mmHg)				MD			MD		
Pulse pressure § (mmHg)				MD			MD		
Heart rate § (bpm)				MD			MD		
Rate pressure product[5]				MD			MD		
Within 24 hours of randomisation									
Systolic BP (mmHg)									

For peer review only

For peer review only - http://bmjopen.bmj.com/site/about/guidelines.xhtml

Mean[1]	MD	MD
Delta[2]	MD	MD
Peak[3]	MD	MD
Standard deviation[4]	MD	MD
Diastolic BP § (mmHg)	MD	MD
Pulse pressure § (mmHg)	MD	MD
Heart rate § (bpm)	MD	MD
Rate pressure product[5]	MD	MD
Neurologic deterioration	OR	OR
Symptomatic hypotension	OR	OR
Within 7 days of randomisation		
Systolic BP (mmHg)		
Mean[1]	MD	MD
Delta[2]	MD	MD
Peak[3]	MD	MD
Standard deviation[4]	MD	MD
Diastolic BP § (mmHg)	MD	MD
Pulse pressure § (mmHg)	MD	MD
Heart rate § (bpm)	MD	MD

For peer review only - http://bmjopen.bmj.com/site/about/guidelines.xhtml

Rate pressure product[5]	MD	MD
Neurologic deterioration	OR	OR
Symptomatic hypotension	OR	OR
**Repeat brain imaging**		
Completed within 48 hours	OR	OR
Haematoma volume		
Absolute (ml)	MD	MD
Expansion		
>33%	OR	OR
>6ml	OR	OR
Perihaematomal oedema volume		
Absolute (ml)	MD	MD
Relative[6]	MD	MD
Mass effect	OR	OR
**90-day clinical outcomes**		
Primary outcome (unfavourable shift in ordinal mRS score)	OR	OR
Death (%)	OR	OR

For peer review only - http://bmjopen.bmj.com/site/about/guidelines.xhtml

Dichotomised mRS score		
4-6, death and major disability (%)	OR	OR
3-6, death and dependency (%)	OR	OR
Barthel index (/100)	MD	MD
EQ-5D-5L (/25)	MD	MD
MMSE (/30)	MD	MD
Serious adverse events		
Fatal	OR	OR
Non-fatal	OR	OR
Renal	OR	OR
Cardiac	OR	OR
Pneumonia	OR	OR
VTE	OR	OR

BP denotes blood pressure; SD, standard deviation; bpm, beats per minute; mRS, modified Rankin Scale; EQ-5D-5L, European Quality of Life–5 Dimensions 5-level questionnaire; MMSE, mini mental state examination; VTE, venous thromboembolism.

[1]Mean of measures within specified period

[2]Baseline minus mean within specified period

[3]Highest value within specified period

[4]Standard deviation of measures within specified period

[5]Peak systolic BP multiplied by peak heart rate within specified period

§Mean, delta, peak and standard deviation will also be assessed

For peer review only - http://bmjopen.bmj.com/site/about/guidelines.xhtml

FIGURE 1. Shift in mRS according to BP lowering vs. control/guideline treatment. Give adjusted odds ratio from generalised linear mixed model with source trial as random effect. Adjusted for age, sex, severity (NIHSS), systolic BP, ICH volume at baseline, IVH at baseline, time to randomisation.

For peer review only

For peer review only - http://bmjopen.bmj.com/site/about/guidelines.xhtml

FIGURE 2. Forest plot of shift in mRS by pre-defined subgroups. Same model as figure 1 with interaction tests.

- Age: ≤70, >70 (years)
- Sex
- Region: Asian vs other
- Pre-stroke hypertension: Y/N
- Pre-stroke antithrombotic use: Y/N
- Baseline SBP: ≤140, 140-180, 180-220, >220 (mmHg)
- NIHSS: ≤ 8, 9-15, >15
- Haematoma volume at baseline: ≤ 15, >15 (ml)
- Type of intervention: target vs angiotensin inhibitor vs CCB vs NO donor vs magnesium/other
- Time from stroke to randomisation: <2, 2-6, 6-48, >48 (hours)
- Type of trial: pre-hospital vs hospital, ICH only vs mixed

For peer review only

For peer review only - http://bmjopen.bmj.com/site/about/guidelines.xhtml

FIGURE 3. Kaplan-Meier curve for death with comparison of active and control treatment groups. Model adjusted for age, sex, severity (NIHSS), systolic BP, ICH volume at baseline, IVH at baseline, time to randomisation and source trial.

For peer review only

For peer review only - http://bmjopen.bmj.com/site/about/guidelines.xhtml

FIGURE 4. Forest plot for end-of-trial death by pre-defined subgroups. Same model as figure 1 with interaction tests.

- Age: ≤70, >70 (years)
- Sex
- Region: Asian vs other
- Pre-stroke hypertension: Y/N
- Pre-stroke antithrombotic use: Y/N
- Baseline SBP: ≤140, 140-180, 180-220, >220 (mmHg)
- NIHSS: ≤ 8, 9-15, >15
- Haematoma volume at baseline: ≤ 15, >15 (ml)
- Type of intervention: target vs angiotensin inhibitor vs CCB vs NO donor vs magnesium/other
- Time from stroke to randomisation: <2, 2-6, 6-48, >48 (hours)
- Type of trial: pre-hospital vs hospital, ICH only vs mixed

For peer review only

FIGURE 5. Individual participant data regression: relationship between randomised treatment group and odds ratio for unfavourable shift in scores on the modified Rankin Scale at end of trial and (i) time to randomisation; (ii) baseline systolic blood pressure; and (iii) baseline haematoma volume. Model (i) is adjusted for age, sex, severity, type of treatment, and source trial; models (ii) and (iii) are adjusted for the same plus time to randomisation.

For peer review only

Reporting checklist for protocol of a systematic review.

Based on the PRISMA-P guidelines.

Instructions to authors

Complete this checklist by entering the page numbers from your manuscript where readers will find each of the items listed below.

Your article may not currently address all the items on the checklist. Please modify your text to include the missing information. If you are certain that an item does not apply, please write "n/a" and provide a short explanation.

Upload your completed checklist as an extra file when you submit to a journal.

In your methods section, say that you used the PRISMA-P reporting guidelines, and cite them as:

Moher D, Shamseer L, Clarke M, Ghersi D, Liberati A, Petticrew M, Shekelle P, Stewart LA. Preferred Reporting Items for Systematic Review and Meta-Analysis Protocols (PRISMA-P) 2015 statement. Syst Rev. 2015;4(1):1.

For peer review only

		Reporting Item	Page Number
Identification	#1a	Identify the report as a protocol of a systematic review	1
Update	#1b	If the protocol is for an update of a previous systematic review, identify as such	n/a
	#2	If registered, provide the name of the registry (such as	5

		PROSPERO) and registration number	
Contact	#3a	Provide name, institutional affiliation, e-mail address of all protocol authors; provide physical mailing address of corresponding author	1
Contribution	#3b	Describe contributions of protocol authors and identify the guarantor of the review	14
	#4	If the protocol represents an amendment of a previously completed or published protocol, identify as such and list changes; otherwise, state plan for documenting important protocol amendments	
Sources	#5a	Indicate sources of financial or other support for the review	14
Sponsor	#5b	Provide name for the review funder and / or sponsor	14
Role of sponsor or funder	#5c	Describe roles of funder(s), sponsor(s), and / or institution(s), if any, in developing the protocol	14
Rationale	#6	Describe the rationale for the review in the context of what is already known	4
Objectives	#7	Provide an explicit statement of the question(s) the review will address with reference to participants, interventions, comparators, and outcomes (PICO)	5
Eligibility criteria	#8	Specify the study characteristics (such as PICO, study design, setting, time frame) and report characteristics (such as years considered, language, publication status) to be used as criteria for eligibility for the review	6

For peer review only

Information sources	#9	Describe all intended information sources (such as electronic databases, contact with study authors, trial registers or other grey literature sources) with planned dates of coverage	7
Search strategy	#10	Present draft of search strategy to be used for at least one electronic database, including planned limits, such that it could be repeated	7
Study records - data management	#11a	Describe the mechanism(s) that will be used to manage records and data throughout the review	7
Study records - selection process	#11b	State the process that will be used for selecting studies (such as two independent reviewers) through each phase of the review (that is, screening, eligibility and inclusion in meta-analysis)	7
Study records - data collection process	#11c	Describe planned method of extracting data from reports (such as piloting forms, done independently, in duplicate), any processes for obtaining and confirming data from investigators	8
Data items	#12	List and define all variables for which data will be sought (such as PICO items, funding sources), any pre-planned data assumptions and simplifications	8, sup. material 2
Outcomes and prioritization	#13	List and define all outcomes for which data will be sought, including prioritization of main and additional outcomes, with rationale	9
Risk of bias in	#14	Describe anticipated methods for assessing risk of bias of	10

For peer review only

individual studies		individual studies, including whether this will be done at the outcome or study level, or both; state how this information will be used in data synthesis	
Data synthesis	#15a	Describe criteria under which study data will be quantitatively synthesised	10
	#15b	If data are appropriate for quantitative synthesis, describe planned summary measures, methods of handling data and methods of combining data from studies, including any planned exploration of consistency (such as I2, Kendall's τ)	11, sup. material 3
	#15c	Describe any proposed additional analyses (such as sensitivity or subgroup analyses, meta-regression)	11, sup. material 3
	#15d	If quantitative synthesis is not appropriate, describe the type of summary planned	n/a
Meta-bias(es)	#16	Specify any planned assessment of meta-bias(es) (such as publication bias across studies, selective reporting within studies)	11
Confidence in cumulative evidence	#17	Describe how the strength of the body of evidence will be assessed (such as GRADE)	11

The PRISMA-P checklist is distributed under the terms of the Creative Commons Attribution License CC-BY 4.0. This checklist was completed on 08. May 2018 using http://www.goodreports.org/, a tool made by the EQUATOR Network in collaboration with Penelope.ai
